# Enhanced Terahertz Photoresponse via Acoustic Plasmon Cavity Resonances in Scalable Graphene

**DOI:** 10.1021/acsphotonics.6c00272

**Published:** 2026-06-01

**Authors:** Domenico De Fazio, Sebastián Castilla, Karuppasamy P. Soundarapandian, Tetiana Slipchenko, Ioannis Vangelidis, Simone Marconi, Riccardo Bertini, Vlad Petrica, Yang Hao, Alessandro Principi, Elefterios Lidorikis, Roshan K. Kumar, Luis Martín-Moreno, Frank H. L. Koppens

**Affiliations:** † Department of Molecular Science and Nanosystems, 19047 Ca’ Foscari University of Venice, Via Torino 155, 30172 Venice, Italy; ‡ The Barcelona Institute of Science and Technology, 172281 ICFO—Institut de Ciències Fotòniques, Avinguda Carl Friedrich Gauss 3, 08860 Castelldefels (Barcelona), Spain; § Instituto de Nanociencia y Materiales de Aragón (INMA), CSIC–Universidad de Zaragoza, 50009 Zaragoza, Spain; ∥ Departamento de Física de la Materia Condensada, Facultad de Ciencias, Universidad de Zaragoza, 50009 Zaragoza, Spain; ⊥ Department of Materials Science and Engineering, 37796 University of Ioannina, GR-451 10 Ioannina, Greece; # School of Electronic Engineering and Computer Science, 4617 Queen Mary University of London, E1 4FZ London, U.K.; ¶ Department of Physics and Astronomy, University of Manchester, Oxford Road, M13 9PL Manchester, U.K.; ∇ Catalan Institute of Nanoscience and Nanotechnology (ICN2), BIST and CSIC, Campus UAB, 08193 Bellaterra (Barcelona), Spain; ○ ICREA—Institució Catalana de Recerca i Estudis Avançats, 08010 Barcelona, Spain

**Keywords:** CVD graphene, acoustic plasmons, terahertz, photothermoelectric effect, photodetectors, Fabry–Pérot cavity, polaritonic photoresponse

## Abstract

Precise control and nanoscale confinement of terahertz (THz) fields are essential requirements for emerging applications in photonics, quantum technologies, wireless communications, and sensing. Here, we demonstrate a polaritonic cavity-enhanced THz photoresponse in an antenna-coupled device based on chemical-vapor-deposited (CVD) monolayer graphene. The dipole antenna lobes simultaneously serve as two gate electrodes, concentrate the impinging THz field, and efficiently launch acoustic graphene plasmons (AGPs), which drive a strong photothermoelectric (PTE) signal. Between 6 and 90 K, the photovoltage exhibits pronounced peaks, modulating the PTE response by up to ∼40%, which we attribute to AGPs forming a Fabry–Pérot THz cavity in the full or half graphene channel. Combined full-wave and transport–thermal simulations accurately reproduce the gate-controlled plasmon wavelength, spatial absorption profile, and the resulting nonuniform electron heating responsible for the PTE response. The lateral and vertical maximum confinement factors of the AGP wavelength relative to the incident wavelength are 165 and 4000, respectively, for frequencies from 1.83 to 2.52 THz. These results demonstrate that wafer-scalable CVD graphene, without hexagonal boron nitride encapsulation, can host coherent AGP resonances and exhibit an efficient polaritonic-enhanced photoresponse under appropriate gating, antenna coupling, and AGP cavity design, opening a route to scalable, polarization- and frequency-selective, liquid-nitrogen cooled, and low-power consumption THz detection platforms based on polaritonic-thermoelectric transduction.

## Graphical abstract

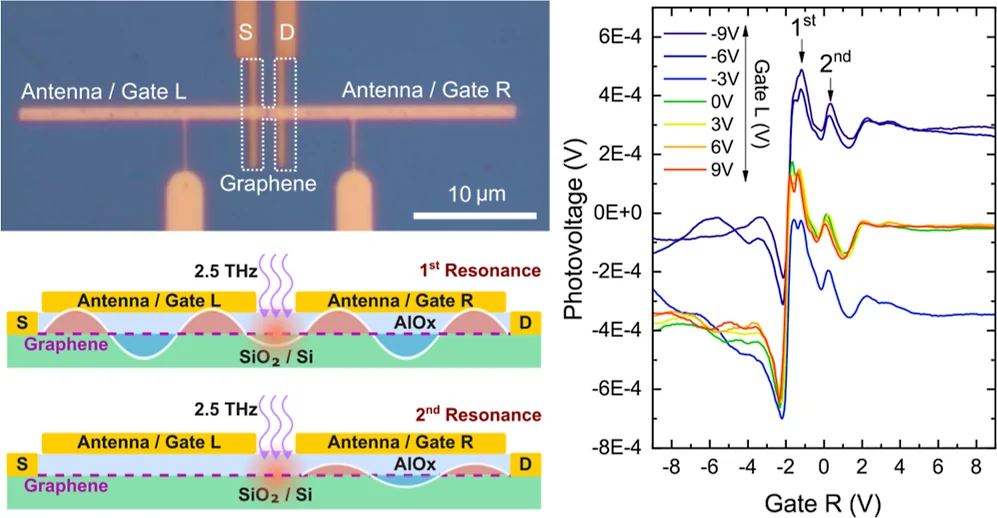

## Introduction

Far-infrared or terahertz (THz) radiation, spanning wavelengths between 15 and 1000 μm, is becoming increasingly important across disciplines due to its unique ability to probe matter noninvasively and with high spectral specificity.^1^ It has found applications in biomedical imaging,^2^ security screening,^3^ high-speed wireless communications,^4^ and chemical sensing,^5^ leveraging its nonionizing nature and ability to interact with low-energy excitations in materials. The growing demand for THz technology has spurred significant advancements in both photonic^6,7^ and electronic^8,9^ device platforms.

A variety of technologies have been developed to detect THz radiation,^10,11^ each with strengths and limitations in terms of sensitivity, speed, and complexity. Among the most common ones, Schottky diode detectors offer room-temperature operation and fast (∼ns) response,^12–15^ but their noise performance degrades significantly above 1–2 THz.^11^ Quantum well infrared photodetectors and quantum cascade detectors can provide fast, frequency-selective detection^16–18^ but are constrained by narrow bandwidth and the need for cryogenic operation.^10^ Thermal detectors, including cryogenically cooled superconducting bolometers, uncooled microbolometers, Golay cells, thermopiles, and more, are among the most sensitive and broadband THz detectors^10,19^ but are typically slow (∼ms) and often require thermal isolation or active cooling.^20,21^

Graphene offers a compelling platform for THz detection due to the combination of broadband absorption, high carrier mobility, electrostatic tunability, low specific heat, and weak electron–phonon coupling.^22,23^ As a gapless two-dimensional semimetal, graphene supports interaction with THz radiation across a wide frequency range, while encapsulation with the atomically flat hexagonal boron nitride (hBN) preserves mobility and screens substrate scattering.^24,25^ Detection schemes such as the photothermoelectric effect (PTE),^24,26,27^ Dyakonov–Shur (DS) rectification,^28^ ballistic transport,^29^ and tunneling^30,31^ have been experimentally demonstrated to exhibit broadband and fast response at room and cryogenic temperatures.^32,33^ However, graphene’s atomically thin nature limits free-space absorption, requiring engineered strategies to enhance light-matter coupling.^34^

Polaritons offer a powerful strategy to compress electromagnetic waves far below the free-space wavelength and enhance light–matter interactions. These hybrid light–matter quasiparticles, including plasmon polaritons in graphene,^35,36^ phonon polaritons in hBN,^36–38^ and more,^39^ can confine incident radiation to deeply subdiffractional volumes, greatly increasing local field intensity and absorption.^40,41^ Most recently, acoustic graphene plasmons (AGPs) have emerged as a promising alternative to conventional (optical) plasmons: by coupling a graphene layer to a nearby metal or another graphene sheet through a dielectric spacer, the system can host antisymmetric charge oscillations that result in even tighter (THz) field confinement and enhanced absorption.^36,42–45^ These acoustic modes offer new opportunities for efficient THz detection by pushing interaction volumes to the extreme nanoscale.^44^

Several experimental studies have leveraged conventional and acoustic plasmons in graphene THz photodetectors and photocurrent spectroscopy architectures.^25,46–48^ For example, Cai et al. demonstrated that patterning graphene into microribbons can introduce a tunable plasmonic resonance at THz frequencies, thereby increasing absorption and enabling PTE THz detection at room temperature.^46^ Alonso-González et al. directly visualized acoustic plasmons in a split-gated graphene photodetector by near-field photocurrent mapping, confirming the expected λ_p_ ≪ λ_0_ (approximately λ_0_/66) and the linear dispersion of AGPs due to coupling with the metal gate.^47^ Bandurin et al. achieved resonant far-field THz detection by using a high-mobility hBN-encapsulated bilayer graphene channel as a plasmonic Fabry–Pérot cavity, observing multiple gate-tunable photocurrent peaks when the channel length accommodated an integer number of plasmon half-waves.^25^ Notably, the resonances were visible only under cryogenic conditions (∼10 K), whereas at room temperature or lower THz frequencies, the plasmonic resonances were overdamped and faded in the noise. In a recent study, a graphene-based THz detector demonstrated plasmonic resonances up to room temperature.^48^ However, these resonances appeared only as weak features in the photoresponse^48^ and therefore did not demonstrate their full potential for the aforementioned applications.

The above-mentioned works underscore that achieving a far-field resonant AGP THz response in scalable chemical-vapor-deposited (CVD) single-layer graphene without hBN encapsulation has not yet been realized. In addition, an efficient method to exploit graphene plasmons to enhance the far-field photoresponse in the terahertz range remains elusive. These challenges motivate the exploration of such a platform for AGP-enhanced THz detectors.

In this work, we demonstrate resonant polaritonic cavity-enhanced THz photoresponse in a split-gated architecture based on CVD single-layer graphene. The gate electrodes are shaped to function as a dipole antenna, designed to efficiently couple 1.83 to 2.52 THz radiation into the graphene channel, act as an AGP launcher, and asymmetrically dope graphene to harness the PTE effect. When cooled to cryogenic temperatures (down to 5 K), we observe pronounced resonances that strongly modulate the photovoltage response as a function of the gate voltage, consistent with AGPs forming standing wave modes in the device. The observed features indicate Fabry–Pérot-type resonances both across the full graphene channel and localized beneath one of the two gates (half channel). These resonances vanish at 130 K while warming the sample up.

So far, graphene plasmonic resonances have typically appeared only as small perturbations in the photovoltage, at least one to 2 orders of magnitude smaller than the conventional photoresponse.^25,48^ Here, we overcome this issue and demonstrate a plasmonically enhanced photoresponse that is 30% higher than the maximum conventional PTE response. We have designed the AGP resonances to align closely in the Fermi energy with the peak of the Seebeck coefficient to maximize the polaritonic cavity-enhanced photoresponse. This shows that our THz AGP cavity geometry is efficiently exploited to enhance the photoresponse and pave the way for further improvements. In contrast to the DS mechanism, in which resonances occur along the entire channel,^25,48^ our cavity geometry defines the AGP resonance within the region where the antenna lobe overlaps with the graphene channel.

The lateral and vertical maximum confinement factors^36,43,49^ of the AGP wavelength relative to the incident wavelength are 165 and 4000, respectively, for frequencies (*f*) from 1.83 to 2.52 THz. Our findings establish that CVD-grown graphene, without the need for hBN encapsulation, can support coherent AGP resonances under suitable architectures, gating conditions, and liquid nitrogen cooling. By further improving the mobility of CVD graphene through novel growth approaches,^50,51^ one can expect the AGP resonances to be maintained up to room temperature. This opens a pathway toward scalable, resonantly enhanced THz photodetectors based on AGPs, with potential applications in polarization- and frequency-selective imaging, spectroscopy, communications, and low-power on-chip THz sensing.

## Results and Discussion

### Device Concept and Simulations

Figure 1a shows a microscope top-view image of our device, while Figure 1b is a sketched side view. Details of the device fabrication steps are discussed in the Experimental Section. CVD monolayer graphene was grown on copper foil and subsequently wet-transferred onto a highly resistive silicon (Si) substrate (ρ > 10, 000 Ω·cm) with a ∼300 nm thermally grown silicon dioxide (SiO_2_) layer. The source (S) to drain (D) electrode separation, i.e., the graphene channel length, is *L* = 2 μm, matching the channel width of *W* = 2 μm. A–40 nm-thick aluminum oxide (AlO_*x*_) layer, deposited by atomic layer deposition (ALD), serves as the gate dielectric. The graphene was patterned into an H-shaped channel using reactive ion etching, a geometry chosen to optimize the contact resistance between graphene and the metallic electrodes.^32^ The split-gate electrodes, designed as the two symmetric half-lobes of a dipole antenna, serve a dual role: they act as metallic dipole arms resonant near 2.5 THz and simultaneously define the graphene channel cavity where AGPs form. The dipole arms are designed to approximate a half-wavelength resonance at 2.5 THz, yielding a capacitive near-field impedance that couples efficiently to the low-impedance plasmonic mode in graphene. In this configuration, the antenna converts incident far-field THz radiation into a strongly localized electric field concentrated across the dipole antenna gap, providing efficient near-field excitation of the AGP modes. As described in the following sections, the spatial overlap between the antenna lobes and the graphene channel defines the AGP THz cavity. The gap between the two gates/antenna lobes is 200 nm, chosen based on the resolution limits of our electron-beam lithography (EBL) system and to avoid leakage between the antenna branches. The total antenna length is 40 μm, optimized through full-wave electromagnetic simulations (see Supporting Information, Figure S2). This design enables simultaneous optical coupling and electrostatic control of the graphene channel, facilitating the exploitation of the PTE effect.^26^

**Figure 1:**
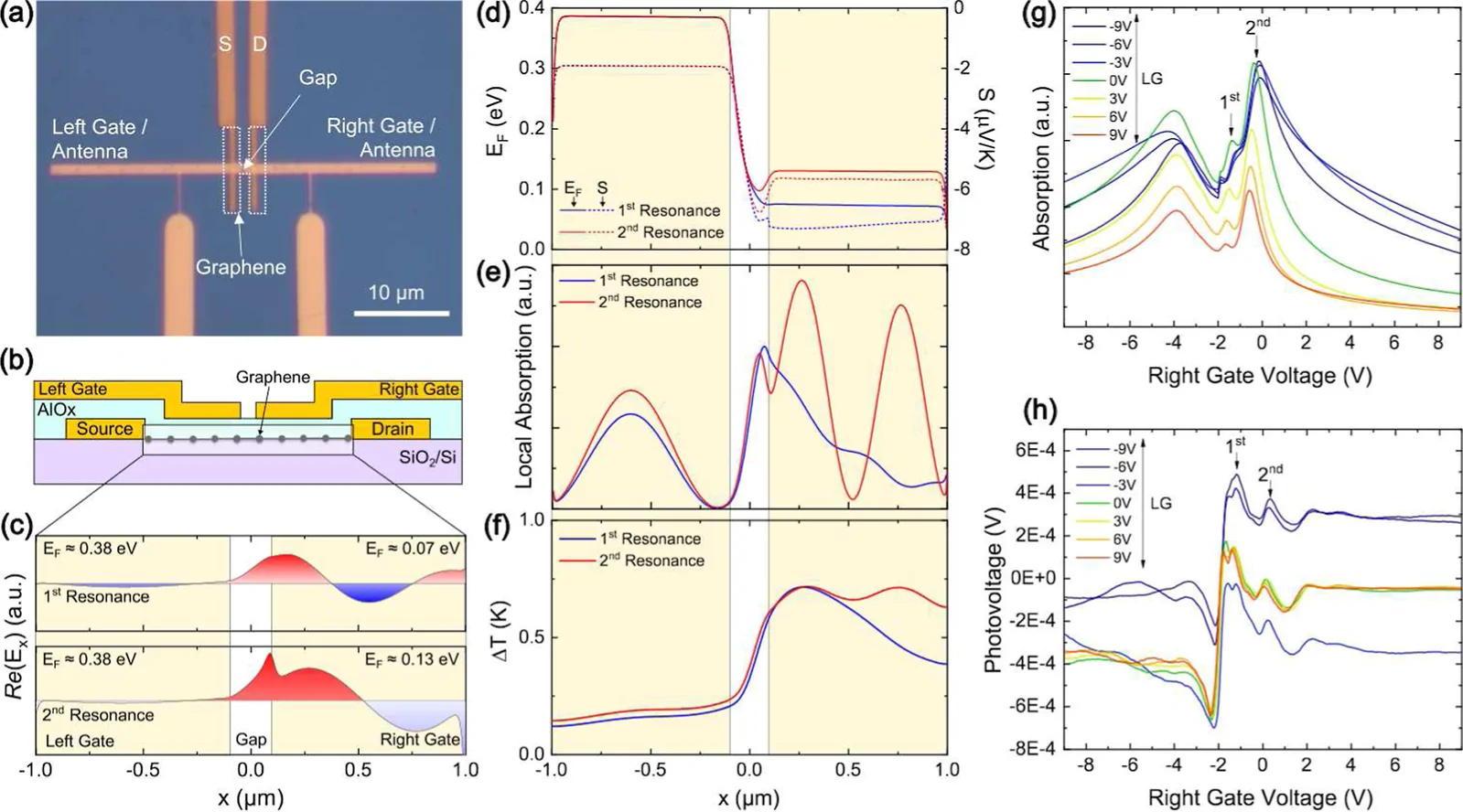
(a) Optical microscope top-view image of the split-gated CVD graphene device. The left and right gates are shaped as a dipole antenna with a 200 nm gap, while the graphene channel (H-shaped, dashed region) connects the source (S) and drain (D) electrodes. (b) Schematic cross-sectional view of the device, showing the AlO_*x*_ gate dielectric, graphene channel, SiO_2_/Si substrate, and the split-gate configuration. (c) Simulated spatial distribution of the real part of the in-plane electric field, Re­(*E*_*x*_), along the graphene channel. The results are shown for the representative left-gate (LG) voltage value of 9 V and two right-gate (RG) voltages, −1.6 V and −0.6 V, labeled as the first and second resonance, respectively. *T* = 6 K, as in (d-g). (d) Simulated Fermi energy distribution *E*_*F*_(*x*) (solid lines) and Seebeck coefficient *S*(*x*) (dotted lines) across the channel for the first (blue) and second (red) gating voltage, i.e., −1.6 V and −0.6 V, highlighting the carrier density modulation induced by asymmetric gating. (e) Calculated local optical absorption profile for the same gate voltages, showing enhanced absorption in the gap region and under the right gate. (f) Simulated electronic temperature increase Δ*T* along the channel for the two resonances, illustrating localized hot-electron formation in regions of high field confinement. (g) Simulated total optical absorption as a function of RG voltage for several fixed LG voltages, revealing gate-tunable AGP resonances (the bidirectional arrow highlights the used LGs). Illumination frequency is 2.5 THz. The electron and hole mobilities under the left and right antenna gates were chosen in agreement with values extrapolated from fits of experimental data in Figure S1; under the antenna gap, the mobility was assumed to vary linearly between these values. (h) Experimentally measured photovoltage at 2.5 THz and 18 mW incident power as a function of RG voltage at several fixed LG voltages, showing resonant features consistent with AGP Fabry–Pérot standing waves in the graphene channel.

The simulated spatial distribution of the real part of the in-plane electric field, Re­(*E*_*x*_), shown in Figure 1c, corresponds to a gating configuration used in the experiment: the LG is kept at *V*_LG_ = 9 V, while the right-gate (RG) voltage *V*_RG_ is swept. We show results for two values, *V*_RG_ = −1.6 V and *V*_RG_ = −0.6 V, which coincide with the two experimentally observed photovoltage peaks hereafter labeled as the first and second resonances, respectively. In both panels, the yellow shading marks the regions beneath the metallic gates, while the central white area denotes the 200 nm antenna gap in the *L* = 2 μm-long graphene channel. At the first resonance (top panel), the field oscillations extend across the entire graphene channel. The distribution exhibits a sequence of nodes and antinodes forming a clear standing-wave pattern. The resonance profile is characterized by two negative lobes [blue, Re­(*E*_*x*_) < 0], one strong positive lobe [red, Re­(*E*_*x*_) > 0], and a truncated positive half-lobe near the right boundary, indicating a slight deviation from an ideal symmetric standing wave. The smooth evolution of the field from the LG region, through the gap, and into the RG region indicates that the full channel length acts as the cavity, i.e., the cavity length *L*_c_ = *L*. Using the Fabry–Pérot resonance condition for nearly ideal mirrors

1 $$\lambda_{p}^{eff}=\frac{2L_{c}}{m}$$

with *L*_c_ = *L* and mode index *m* = 4, we obtain an effective plasmon wavelength λ_p_^eff^ ≈ *L*/2. The strong AGP reflection at the gold contacts justifies the assumption of nearly ideal mirrors. In contrast, at the second resonance (bottom panel), the field distribution exhibits a qualitatively different behavior. The oscillation pattern beneath the LG closely resembles that of the first resonance, as expected, since in both cases, the left region is tuned to the same Fermi energy *E*_F_ by *V*_LG_ = 9 V. However, the overall field profile is markedly different: we observe an abrupt transition in the gap region and truncated field amplitudes near both contacts. This abrupt change originates from a sharp variation in carrier density—and thus surface conductivity—within the gap region (see the *E*_F_ distributions in Figure 1d), which leads to strong plasmon backscattering, in agreement with previous work.^52^ By contrast, for the first resonance, the conductivity profile varies more smoothly across the gap, so that AGP reflection at this interface is strongly reduced and plasmon backscattering is suppressed. In the second-resonance configuration, the abrupt conductivity change at the gap edge, together with the metal contacts, effectively turns the graphene channel into two subcavities of length *L*_c_ = *L*/2, each bounded by a metal contact at one end and by the gap region at the other. In principle, each subcavity can support its own plasmonic modes. In the right subcavity, the field develops an approximate node–antinode–node–antinode sequence, which can again be described by the Fabry–Pérot condition.

2 $$\lambda_{p,R}=\frac{2L_{c}}{m}$$

with *L*_c_ = *L*/2 and *m* = 2, yielding λ_p,*R*_ ≈ *L*/2. This appears as alternating red and blue lobes emerging from the gap edge. The similarity of the oscillatory pattern under the LG at both resonances, together with the clear formation of two subcavities, indicates that the experimentally observed second photovoltage peak is primarily associated with a Fabry–Pérot AGP mode confined in the right subcavity. Altogether, these oscillatory field profiles provide direct evidence of Fabry–Pérot–type AGP modes, with the first resonance corresponding to a mode extending across the full channel and the second resonance corresponding to a mode localized mainly beneath the right gate. The distinct behavior observed at the two RG voltages will be analyzed in more detail in the following sections.

The oscillatory field distributions in Figure 1c originate from the asymmetric carrier-density profile along the channel. By applying different voltages to the left and right gates, a nonuniform carrier concentration profile *n*(*x*) is induced in the graphene channel. Since the Fermi energy is directly related to the local carrier density through

$$E_{F}\left(x\right)=sgn\left(n\left(x\right)\right)\hbar v_{F}\sqrt{\pi \left|n\left(x\right)\right|}$$

the gating asymmetry results in significantly different *E*_F_(*x*) values beneath the two gates. The corresponding Fermi-energy distributions are shown in Figure 1d for the same gate-voltage configurations as in Figure 1c. The spatial modulation of *E*_F_(*x*) directly determines the local optical absorption of the graphene channel, which can be expressed as *A*(*x*) ∝ Re­[σ­(*E*_F_(*x*))] |*E*_*x*_(*x*)|^2^, where σ­(*E*_F_(*x*)) is the graphene optical conductivity, which is spatially dependent on the Fermi energy. The local absorption is illustrated in Figure 1e for the two gate-voltage configurations discussed above. For both resonances, absorption is strongly enhanced inside the antenna gap. For the first resonance (blue curve), the gap peak is followed by a weaker extension beneath the right gate and a smaller, smoother peak beneath the left antenna. In contrast, for the second resonance (red curve), the maximum absorption peak is larger, with the gap peak followed by multiple pronounced maxima under the right gate and a weaker one beneath the LG. These spatially localized absorption hotspots provide the direct source of electronic heating: the absorbed optical power is dissipated into the electronic system, producing the nonuniform electron–temperature profiles *T*_e_(*x*) shown in Figure 1f.^38^ The local temperature rise relative to equilibrium, Δ*T*(*x*) = *T*_e_(*x*) – *T*, closely follows the absorption pattern. For the first resonance (blue), the temperature rise is sharply peaked just to the right of the gap. For the second resonance (red), the heating profile is broader and more extended, with maxima shifted farther into the gated region. This spatially varying *T*_e_(*x*) generates a thermoelectric voltage according to *V*_PTE_ ∝ *S*(*x*)∇*T*_e_(*x*), where *S*(*x*) denotes the Seebeck coefficient determined by the local Fermi energy through the Mott relation^53^

3 $$S\left(x\right)=-\frac{\pi^{2}k_{B}^{2}T_{e}\left(x\right)}{3e}\frac{1}{\sigma \left(E_{F}\left(x\right)\right)}\frac{\partial \sigma \left(E_{F}\left(x\right)\right)}{\partial E_{F}\left(x\right)}$$

where *k*_B_ is the Boltzmann constant, *e* is the elementary charge, σ­(*x*) is the local electrical conductivity, and *E*_F_(*x*) is the local Fermi energy. For the quantitative simulations of the *T*_e_(*x*) profiles, we used our full thermoelectric framework, in which *S*(*x*) is evaluated in its energy-resolved form via Boltzmann/Onsager transport (see Methods of ref 54). The spatial variation of *S*(*x*) is shown by the dotted curves in Figure 1d. The values of *S*(*x*) are smaller at cryogenic temperatures compared to those at room temperature, as expected from the Mott relation.^55^

It is well established that the response of the photodetector can be captured, to a good approximation, by the simulated total absorption of graphene, because the locally absorbed power determines the nonuniform electron–temperature profile *T*_e_(*x*), which drives the thermoelectric voltage through *V*_PTE_ ∝ *S*(*x*)∇*T*_e_(*x*).^38,56^ In Figure 1g, we present the calculated total absorption (integral of local absorption across the entire channel) of graphene for several fixed LG voltages while tuning the RG voltage. The spectra exhibit three prominent absorption peaks: two on the electron-doped side of the right gate charge neutrality point (CNP, right side), whose positions remain nearly invariant with *V*_LG_, and one on the hole-doped side (left side). The CNP is assumed to be at *V*_RG_ ∼−2 V for the right gate and at *V*_LG_ ∼−4 V for the LG, as per the experimental measurements. The difference in the CNP found in the experiments when sweeping each of the gates is likely due to fabrication process residues and local inhomogeneities. Furthermore, previous work has shown that ALD AlO_*x*_ on graphene can drive the material into *n*-doping by virtue of charge transfer at the graphene/oxide interface.^57^ The peak amplitudes depend strongly on *V*_LG_: more negative LG voltages (blue curves) result in stronger absorption, whereas more positive LG voltages (yellow curves) suppress the response. All three peaks originate from AGP mode excitations. For *V*_LG_ = 9 V, the first and second resonances on the electron-doped side are located exactly at *V*_RG_ = −1.6 V and *V*_RG_ = −0.6 V, respectively. For other LG voltages, the resonance positions remain very close to these values. This explains the choice of RG voltages used for the profiles shown in Figure 1c–f.

### Optoelectronic Measurements

Figure 1h shows the measured photovoltage *V*_ph_ as a function of the RG voltage *V*_RG_ for several fixed values of the LG voltage *V*_LG_ at a temperature of *T* = 6 K. The data reveal a rich structure dominated by a prominent polarity inversion near the graphene CNP and multiple resonant features that we attribute to the excitation of acoustic AGPs. A sharp sign reversal of the photovoltage occurs around *V*_RG_ ≈ −2 V, which corresponds to the CNP of the RG graphene region. This feature, together with the strong peak at *V*_RG_ just above *V*_CNP_ (i.e., at ≈ −1.6 V), followed by a slowly decaying tail, is a hallmark of the PTE effect in graphene-based devices.^26,58^ This peak in photovoltage, *V*_ph_, arises from the steep variation of *S* with carrier density between the two asymmetrically gated graphene regions under the two branches of the dipole antenna, combined with the large photoinduced Δ*T* localized in the antenna gap.

At gate voltages above the CNP, two distinct resonant peaks are visible in Figure 1h at ≈ −1.2 V and ≈ 0.3 V, labeled as the first and second resonances, respectively. These are very close to the theoretically predicted values of ≈ −1.6 V and ≈ −0.6 V and correspond to the standing-wave modes of AGPs predicted by simulations. The small offset between the experimental photovoltage peaks and the simulated absorption maxima, particularly visible for the second resonance, may be attributed to the simplified model assumptions of the graphene region below the antenna gap, which has a major role in the half-channel resonance.^38^ The positions of the resonances evolve moderately with *V*_LG_, indicating that the resonances are primarily controlled by the carrier density under the right gate, while the LG mostly tunes the global carrier asymmetry required for PTE detection. These peaks coincide with enhanced local absorption in the graphene channel, as confirmed by the simulated absorption profile in Figure 1e, demonstrating a direct link between plasmon-enhanced field confinement and the observed photovoltage response. This, together with the frequency, incident polarization, and power dependences, is inconsistent with nonplasmonic alternatives such as static inhomogeneous heating or device-specific geometric asymmetries, which would not produce gate-tunable resonant features. In addition to these two well-pronounced features, a third resonance appears at V_RG_ < CNP. This resonance is discernible in the experimental spectra but is significantly weaker than predicted by the simulations. The origin of this reduced visibility is not fully clear, but it may be related to increased disorder or other asymmetries under negative bias that are not captured by the model. Furthermore, it is important to distinguish the antenna resonance from the plasmonic cavity resonances: while the metallic dipole antenna provides broadband coupling around its fundamental resonance (∼2.5 THz), the pronounced photovoltage peaks in Figure 1h arise from AGP Fabry—Pérot resonances confined within the channel. In this picture, the antenna primarily serves as an efficient launcher and receiver of these localized plasmons, rather than setting the overall device resonance. In the following, we therefore focus our analysis on the two resonances above the CNP, which provide the clearest and most reproducible signatures of AGPs.

The dependence on *V*_LG_ also reveals subtle features: at increasingly negative *V*_LG_ (from −3 to −9 V, blue traces), the overall magnitude of *V*_ph_ increases, consistent with stronger PTE contributions arising from larger Seebeck asymmetry between the two gated regions. At the same time, several smaller satellite peaks appear at higher carrier densities. These may originate from higher-order plasmonic overtones or weak cavity-assisted effects; however, their amplitudes are much lower than the primary first and second AGP resonances, and thus, they are not analyzed in this study.

Figure 2 provides a comprehensive view of the gate-dependent resistance and photovoltage response, highlighting the interplay between PTE effects and resonant excitation of AGPs. Figure 2a shows the two-dimensional resistance map *R*(*V*_LG_, *V*_RG_) measured at *T* = 6 K using a constant source-drain bias current of *I*_bias_ = 200 nA (see Experimental Section for details on the measurement setup). The map exhibits the characteristic four-quadrant symmetry typical of dual-gated graphene devices.^26^ The central bright region of enhanced resistance corresponds to the CNP region, where the carrier density beneath the two gates approaches zero. The CNP is at *V*_RG_ ∼−2 V and *V*_LG_ ∼−4 V. Moving away from the CNP along either gate axis, *R* decreases due to electrostatic doping of the graphene. Next, we illuminate the device with a continuous-wave 2.5 THz laser at *T* = 6 K and display the corresponding photovoltage map *V*_ph_(*V*_LG_, *V*_RG_) in Figure 2b. Unlike the 4-fold symmetry of *R*, the photovoltage shows a 6-fold pattern, with six alternating regions of positive (red) and negative (blue) photovoltage values. This six-quadrant symmetry is another hallmark of the PTE effect in graphene-based THz photodetectors,^26,58,59^ arising from the opposite signs of the Seebeck coefficient *S* across different carrier-type configurations, and confirms that the broad background photoresponse (away from the AGP resonance conditions) follows the expected PTE behavior. Superimposed on this PTE background are additional finer modulations of *V*_ph_, seen as oscillatory ripples running parallel to the *V*_RG_ axis in Figure 2b. These correspond to the resonant excitation of AGPs, in agreement with simulations in Figure 1c-g, and consistent with the sharp peaks observed in Figure 1h at *V*_RG_ ≈ −1 V (first resonance) and *V*_RG_ ≈ 0.3 V (second resonance). The comparison between Figures 2b and 1e confirms that these photovoltage resonances coincide with enhanced optical absorption, establishing a direct link between localized field confinement and photoresponse.^36,38^

**Figure 2:**
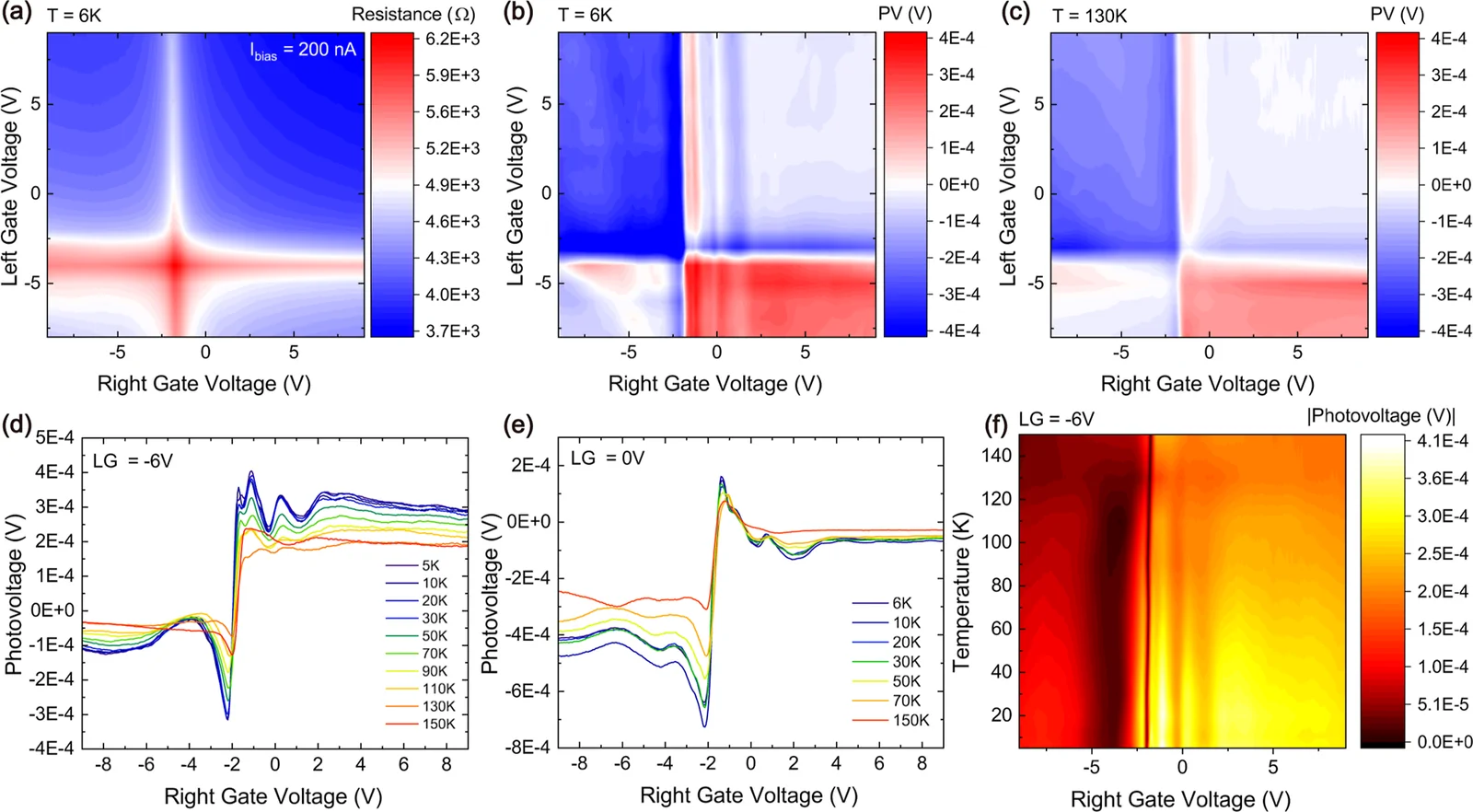
Gate- and temperature-dependent resistance, photovoltage, and AGP resonances. (a) Two-dimensional resistance map as a function of left- and right-gate voltages at *T* = 6 K, measured with *I*_bias_ = 200 nA. (b) Corresponding photovoltage *V*_ph_ map at *T* = 6 K under *f* = 2.5 THz illumination, showing the characteristic 6-fold polarity pattern of the PTE effect and sharp resonant features associated with AGPs. (c) Same measurement at *T* = 130 K, where AGP resonances are strongly suppressed by increased plasmon damping. (d,e) Line cuts of *V*_ph_ vs *V*_RG_ for several temperatures at fixed *V*_LG_ = −6 V (d) and *V*_LG_ = 0 V (e), revealing two prominent AGP resonances around *V*_RG_ ≈ −1.2 and 0.3 V that gradually weaken with increasing *T* and are enhanced when Seebeck asymmetry is maximized. (f) False-color map of |*V*_ph_| vs *V*_RG_ and temperature at *V*_LG_ = −6 V, highlighting the progressive damping and slight redshift of the AGP resonances with increasing *T*.

At elevated temperatures (*T* = 130 K, Figure 2c), the AGP resonances vanish, while the overall six-quadrant PTE symmetry persists. This disappearance is attributed to enhanced electron–phonon scattering, which reduces the carrier mobility μ and increases the intrinsic damping of collective charge excitations, resulting in a broadened plasmon line width γ and a reduced plasmon quality factor *Q*.^25,60^ *Q* indeed provides a direct measure of plasmon losses, expressing the balance between energy storage and dissipation during a plasmon oscillation.^25,60^ From low-temperature transport characterization (see Figure S1), we extract mobilities of μ_*h*_ ≈ 3.7 × 10^4^ cm^2^ V^–1^ s^–1^ for holes and μ_*e*_ ≈ 1.3 × 10^4^ cm^2^ V^–1^ s^–1^ for electrons at *T* = 6 K in the RG region, while μ_*h*_ ≈ 2.7 × 10^4^ cm^2^ V^–1^ s^–1^ and μ_*e*_ ≈ 1.2 × 10^4^ cm^2^ V^–1^ s^–1^ in the LG region. The lower mobility under the LG is attributed to fabrication-induced disorder (see Figure S1), and results in asymmetric plasmon damping along the channel: the plasmon is more strongly attenuated in the LG region, reducing the overlap of the first resonance mode with the LG and affecting the effective quality factor of the full-channel cavity. This disorder-induced asymmetry is an important practical consideration for future device optimizations.

Figure 2d,e further illustrates the temperature dependence of *V*_ph_ while sweeping the RG voltage *V*_RG_ at fixed LG voltages *V*_LG_ = −6 V and *V*_LG_ = 0 V, respectively. At *V*_LG_ = −6 V, the Seebeck asymmetry between the LG and RG regions is maximized, resulting in a strong PTE response and pronounced AGP resonances at *V*_RG_ ≈ −1 and 0.3 V. As the temperature increases from 6 to 130 K, for example, the amplitude of the first resonance is reduced by at least a factor of ∼7, reflecting the progressive increase of intrinsic plasmon damping with temperature. A more quantitative analysis of the peak-to-background contrast, based on the normalized modulation intensity *M*_*i*_(*T*) of each resonance (see Figure S3), shows that the first AGP mode modulates the photovoltage by *M*_1_ ≈ 40% at *T* = 6 K, decreasing to ≲ 15% at *T* ≈ 130 K, while the second mode evolves from *M*_2_ ≈ 25–30% to below ∼5% over the same temperature range. This pronounced reduction of *M*_*i*_(*T*) closely follows the expected temperature dependence of *Q*, governed by phonon- and dielectric-limited damping mechanisms.^61^ While carrier mobility provides a useful qualitative indicator of loss,^62^ the lifetime of graphene plasmons is more directly determined by the imaginary part of the normalized plasmon wavevector *q*_p_ = *k*_p_/*g*, which can be accessed within our electromagnetic simulations. In the acoustic plasmon regime, the plasmon attenuation obeys Im­(*q*_*p*_) = Re­(*q*_*p*_)/(ωτ), where τ is the effective carrier scattering time entering the graphene conductivity, and ω = 2π*f* is the angular frequency of the plasmon mode. Assuming the approximately linear dispersion relation of AGPs, ω ≈ *v*_*g*_Re­(*q*_*p*_)*g*, where *v*_*g*_ is the group velocity of the acoustic plasmon mode and *g* = ω/*c* is the free-space wavenumber, the corresponding plasmon lifetime can be expressed as $\tau_{p}={\left[Im\left(q_{p}\right)gv_{g}\right]}^{-1}$ , yielding τ_*p*_ ≈ τ, and the associated quality factor follows as *Q* = ωτ_p_ = ωτ. This framework highlights that the observed AGP resonances are primarily limited by intrinsic graphene losses, with geometric confinement mainly defining the cavity mode structure rather than the damping rate itself.

Applying this analysis to our simulated structures, and since τ_*p*_ ≈ τ, we estimate the plasmon lifetime from the carrier scattering time $\tau =\mu \left|E_{F}\right|/v_{F}^{2}$ (*v*_*F*_ = 1.15 × 10^6^ m/s), yielding, for the second AGP resonance confined under the right gate in the electron-doped regime (*E*_*F*_ ≈ 0.13 eV, μ_*e*_ ≈ 1.3 × 10^4^ cm^2^ V^–1^ s^–1^), a lifetime τ_*p*_ ≈ 0.13 ps and a corresponding quality factor *Q* ≈ 2.0, whereas for the first resonance, which extends over the full channel and is subject to asymmetric damping (RG: *E*_*F*_ ≈ 0.07 eV, μ_*e*_ ≈ 1.3 × 10^4^ cm^2^ V^–1^ s^–1^; LG: *E*_*F*_ ≈ −0.12 eV, μ_*h*_ ≈ 2.7 × 10^4^ cm^2^ V^–1^ s^–1^), a conservative lower-bound estimate of the plasmon lifetime, obtained by adding the corresponding regional scattering rates as 1/τ_*p*_ = 1/τ_RG_ + 1/τ_LG_, gives τ_*p*_ ≈ 0.05 ps, yielding *Q* ≈ 0.8. These values are in good agreement with estimates from the device parameters of near-field experiments by Alonso-González et al.,^47^ which yield *Q* ≈ 4–12 using the same formula $Q=\omega \tau =\omega \mu \left|E_{F}\right|/v_{F}^{2}$ , reflecting the higher mobility of their hBN-encapsulated exfoliated graphene compared to our nonencapsulated CVD device; they quantitatively reproduce both the resonance visibility and the temperature-induced suppression observed experimentally in Figure 2. We note that *Q* values reported for graphene plasmon resonators at mid-infrared frequencies^44^ are not directly comparable, as *Q* scales linearly with ω. As the temperature increases from 6 to 130 K, the effective reduction of τ_*p*_ inferred from experiment follows the enhanced plasmon damping expected from increased phonon scattering, providing a coherent link between the simulated plasmon losses and the measured temperature dependence.

At *V*_LG_ = 0 V, where the LG region is near its CNP, the Seebeck contrast is minimal, suppressing the overall PTE response and reducing the visibility of the AGP resonances. This confirms that plasmon-enhanced THz detection in our devices relies on the combined effect of (i) strong Seebeck asymmetry to convert absorbed power into measurable photovoltage, and (ii) sufficiently low intrinsic losses in the gated antenna region, for which high carrier mobility provides a useful proxy, to sustain long-lived AGPs. In Figure 2f, we present a color map of the absolute photovoltage |*V*_ph_| as a function of *V*_RG_ and temperature at fixed *V*_LG_ = −6 V, illustrating the thermal evolution of the AGP resonances. As the temperature increases, both resonant features progressively fade and eventually vanish, consistent with enhanced plasmon damping caused by thermally activated carrier scattering and screening effects.^63^

Figure 3 summarizes the tunability and physical origin of the plasmonic resonances in our split-gated graphene device. The experimental data (filled symbols) and theoretical predictions (open symbols) in Figure 3a reveal a systematic evolution of the resonance voltages as the LG bias *V*_LG_ is varied. Two distinct regimes are evident: for *V*_LG_ ≲ −3 V, the resonances follow one trend, whereas for *V*_LG_ ≳ 3 V, the slopes change noticeably. This crossover occurs close to the charge-neutrality point of the graphene under the LG $\left(V_{LG}^{CNP}\approx -4V\right)$ , indicating that the change in doping polarity under the LG plays an important role in determining the resonance behavior. In addition, both the first (blue) and second (red) resonances gradually shift toward lower *V*_RG_ as *V*_LG_ increases, although this effect is comparatively weaker. The excellent agreement between experimental measurements and the AGP cavity model over the entire *V*_LG_ range validates our interpretation of the device as an electrostatically tunable AGP cavity.

**Figure 3:**
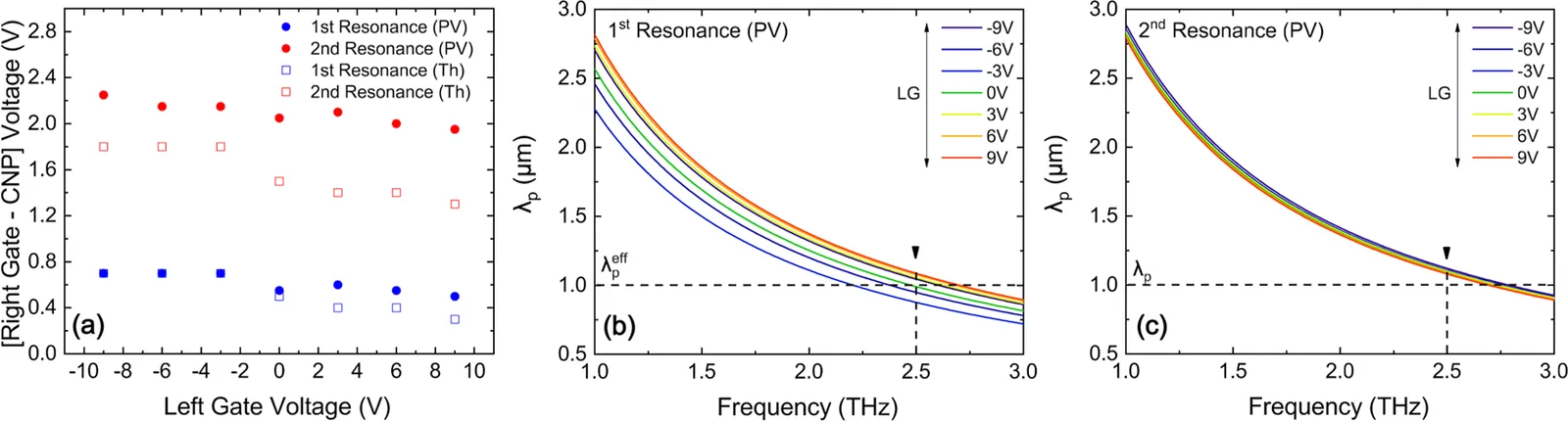
(a) Comparison between measured photovoltage (PV/filled symbols) and simulated absorption (Th/open symbols) peak positions for the first and second AGP resonances in the split-gated graphene device. Blue and red symbols correspond to the first and second resonances, respectively. (b,c) Colored curves correspond to the AGP dispersions calculated at the right-gate voltages corresponding to the first (b) and second (c) resonances extracted from the photovoltage spectra. Horizontal dashed lines mark the Fabry–Pérot conditions: λ_p_^eff^ = *L*/2 for a mode spanning the full channel (b), and λ_p_ = *L*/2 for a mode confined mainly to the right half of the channel, where λ_p_ denotes the local plasmon wavelength under the right gate (c). Vertical dashed lines with arrows indicate the experimental resonance frequency at ∼2.5⁡THz .

To directly relate the measured photovoltage peaks to AGP modes, we compared the experimental data with theoretical predictions based on the AGP dispersion and Fabry–Pérot conditions.^47^ Figure 3b,c shows the AGP dispersion calculated at the RG voltages corresponding to the first and second resonances observed in the photovoltage spectra (see Figure 1h). The colored curves give the plasmon wavelength as a function of frequency *f*, λ_p_(*f*), for different LG voltages *V*_LG_, obtained from the AGP dispersion for a gated graphene sheet^64^

4 $$\lambda_{p}=\frac{16\pi \alpha_{0}E_{F}}{g\hbar \omega \left(\varepsilon_{SiO_{2}}+\sqrt{\varepsilon_{SiO_{2}}^{2}+\frac{16\alpha_{0}cE_{F}\varepsilon_{AlO_{x}}}{\hbar \omega^{2}d}}\right)}$$

where α_0_ is the fine-structure constant, and $\varepsilon_{AlO_{x}}=9.74$ and $\varepsilon_{SiO_{2}}=4.78$ are the permittivities at ∼THz frequencies of the AlO_*x*_ layer of thickness *d* and of the SiO_2_ substrate, respectively.^65^

The horizontal dashed lines in Figure 3b,c represent Fabry–Pérot conditions for two different cavity configurations: a mode spanning the full graphene channel (first resonance, Figure 3b) and a mode mainly confined beneath the right gate (second resonance, Figure 3c). For the full channel of length *L*, the effective plasmon wavelength is given by the harmonic mean of the local wavelengths under the left and right gates^36,49^

5 $$\lambda_{p}^{eff}=\frac{2\lambda_{p,L}\lambda_{p,R}}{\lambda_{p,L}+\lambda_{p,R}}$$

The corresponding Fabry–Pérot resonance condition can be written as

6 $$\lambda_{p}^{eff}=\frac{2L_{c}}{m},\;m=1,2,...$$

with *L*_c_ = *L* for a mode that extends across the entire channel. For the experimentally observed first resonance, we take *m* = 4, in agreement with the four-node standing-wave pattern in the upper panel of Figure 1c. Because the graphene channel is electrostatically inhomogeneous, the local plasmon wavelength differs under the two gates, and the total plasmon propagation is determined by both regions, naturally leading to the harmonic-mean form of λ_p_^eff^. When only the right half of the channel of length *L*/2 contributes, the relevant cavity length is *L*_c_ = *L*/2, and the local plasmon wavelength under the right gate, λ_p,*R*_, satisfies

7 $$\lambda_{p,R}=\frac{2L_{c}}{m},\;m=1,2,...$$

The second resonance is then described by *m* = 2, consistent with the two-node standing-wave pattern observed in the lower panel of Figure 1c. In both cavity configurations, the intersections of the dispersion curves with the dashed lines yield resonance frequencies close to 2.5 THz, in good agreement with the experimental excitation conditions. A comparison of the field profiles in Figure 1c shows that the oscillation pattern under the LG is very similar for the first and second resonances, both in shape and in amplitude. This indicates that the lower mobility in the left part of the channel mainly controls the plasmon damping and local field strength in that region for both resonances, but does not by itself determine whether the dominant cavity spans the full channel or only its right half. The transition from a full-channel Fabry–Pérot mode to a mode localized in the right subcavity is instead primarily governed by the carrier-density distribution in the gap, which sets the surface–conductivity profile and the associated AGP reflection at the gap edge (see Figures 1d and S4). The lower mobility under the LG increases plasmon damping in that region, while the abrupt conductivity change at the gap acts as an additional plasmon reflector; the two effects jointly determine the effective cavity length.

At the second resonance, the sharp conductivity variation in the gap produces strong plasmon backscattering and, together with the metal contacts, effectively defines a right subcavity of length *L*_c_ = *L*/2. The Fabry–Pérot condition is then predominantly sustained by this right subcavity, yielding a local plasmon wavelength λ_p,*R*_ ≈ 1.1 μm, close to *L*/2. By contrast, at the first resonance, the conductivity profile across the gap is much smoother, so AGP reflection at this interface is strongly reduced, and a mode extending across the entire channel is supported. In this case, the effective plasmon wavelength, determined by both halves of the channel, is $\lambda_{p}^{eff}\approx 1.18\mu m$ , close to *L*/2 = 1 μm, as expected for the full-channel Fabry–Pérot condition. Thus, while the mobility asymmetry sets the level of damping in the left region for both resonances, it is the gap-induced conductivity profile that primarily controls whether the AGP mode behaves as a full-channel cavity or as a right subcavity.

To further characterize the device’s performance as a THz photodetector, we investigate the power dependence of the measured photovoltage at *V*_LG_ = −6 V, as shown in Figure 4. In Figure 4a, we control the incident THz power by using a pair of wire-grid polarizers, where one is fixed while the other is rotated to vary the transmitted intensity. A systematic reduction of the resonance amplitude is observed as the relative angle between the polarizers increases, resulting in lower incident power on the device. The two main resonances remain clearly visible even at strongly attenuated powers, demonstrating the robustness of the photoresponse against variations in optical excitation. An independent power dependence study is reported in Figure 4b, where the incident THz power is attenuated with THz absorbers. Consistent with the polarizer-based measurements, the photovoltage amplitude scales monotonically with the incident power, confirming the reliability of the response over 2 orders of magnitude in excitation intensity.

**Figure 4:**
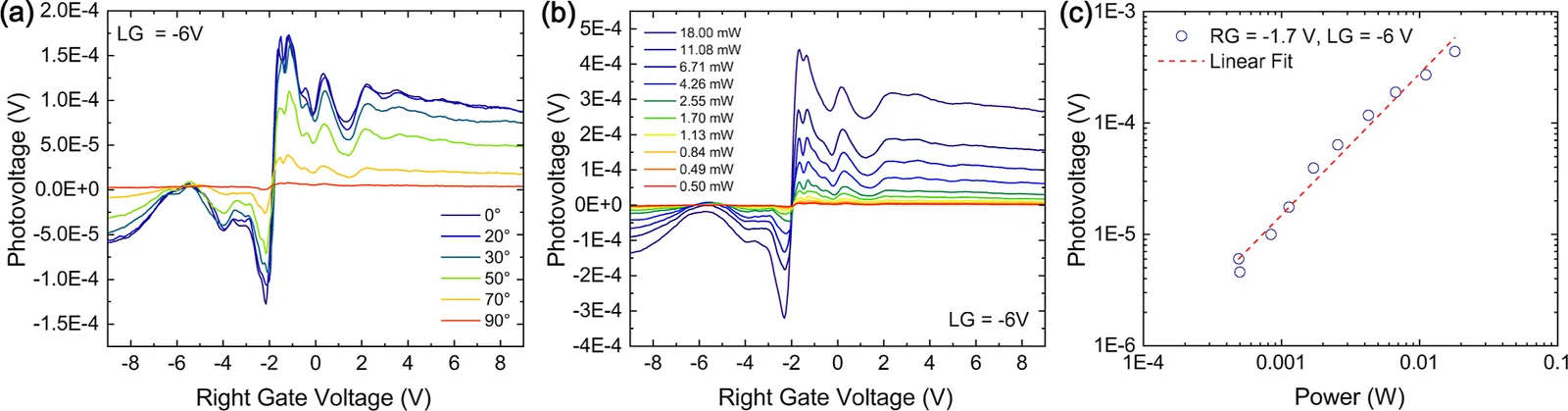
Power dependence of the device photoresponse at *V*_LG_ = −6 V. (a) Measured photovoltage as a function of right-gate voltage *V*_RG_ for different incident powers, controlled using two wire-grid polarizers. (b) Independent power dependence obtained by tuning the output power of the laser source, showing a consistent trend over more than 1 order of magnitude in excitation intensity. (c) Log–log plot of the peak photovoltage as a function of incident power, measured at fixed *V*_RG_ = −1.7 V and *V*_LG_ = −6 V. The nearly linear scaling across almost two decades demonstrates that the device operates in the linear response regime, ensuring predictable and stable performance suitable for THz detection applications.

To quantify the detector performance, we fix the gate voltages at *V*_RG_ = −1.7 V and *V*_LG_ = −6 V, corresponding to the maximum responsivity, and plot the photovoltage as a function of the incident power in Figure 4c. The resulting log–log plot reveals a nearly linear scaling of the photovoltage over 2 orders of magnitude of power, with a fitted slope of ∼1. This linear dependence is a hallmark of a highly efficient and predictable detection regime, confirming that the device operates in the linear response regime across the tested power range. Such behavior is particularly promising for THz detection applications, where stable responsivity over a wide dynamic range is a key figure of merit.

### Frequency Dependence

We now investigate the frequency dependence of the device responsivity at *T* = 6 K, as summarized in Figure 5. The device responsivity is calculated as *R*_v_ = (*V*_ph_/*P*_0_) × (*A*_focus_/*A*_diff_), where *V*_ph_ is the measured photovoltage, *P*_0_ is the incident THz power, A_focus_ is the experimental beam area at the measured wavelength determined from Gaussian beam diameter measurements, and A_diff_ is the diffraction-limited spot size^26,28,32,38^ (see the Experimental Section and Figure S5). In Figure 5a, we compare the measured photovoltage response at two different excitation frequencies, *f* = 2.5 THz (blue curve) and *f* = 1.8 THz (red curve), for a fixed *V*_LG_ = −6 V. Both data sets exhibit the same gate-dependent features, corresponding to the acoustic plasmon resonances previously discussed, while the overall responsivity at 1.8 THz is reduced compared to 2.5 THz. This trend directly reflects the antenna-coupling efficiency, which is expected to peak near the designed dipole resonance at *f* ≈ 2.5 THz. The maximum responsivity at 2.5 THz is ∼12⁡V/W , yielding a noise equivalent power (NEP) of $∼100pW/\sqrt{Hz}$ at 6 K (see the Experimental Section). For context, room-temperature antenna-coupled graphene THz PTE detectors based on hBN-encapsulated graphene have achieved responsivities up to ∼100⁡V/W and NEP values of ∼80– $350pW/\sqrt{Hz}$ ;^26,32^ Caridad et al.^48^ reported ∼1.8⁡V/W at cryogenic temperatures (∼10⁡K) ; Bandurin et al.^25^ demonstrated NEP $∼0.2pW/\sqrt{Hz}$ and responsivity ∼3000⁡V/W at 0.13 THz using high-mobility hBN-encapsulated bilayer graphene. These values are overall comparable to our results, as we note that the different operating temperatures, frequencies, and graphene quality across these works complicate a direct comparison of absolute figures of merit. The key advance of the present work lies not in absolute sensitivity, but in the resonant polaritonic enhancement: the first AGP mode alone modulates the PTE photovoltage by up to ∼40%, achieved in nonencapsulated CVD graphene, which represents a more scalable and manufacturable platform than hBN-encapsulated devices.

**Figure 5:**
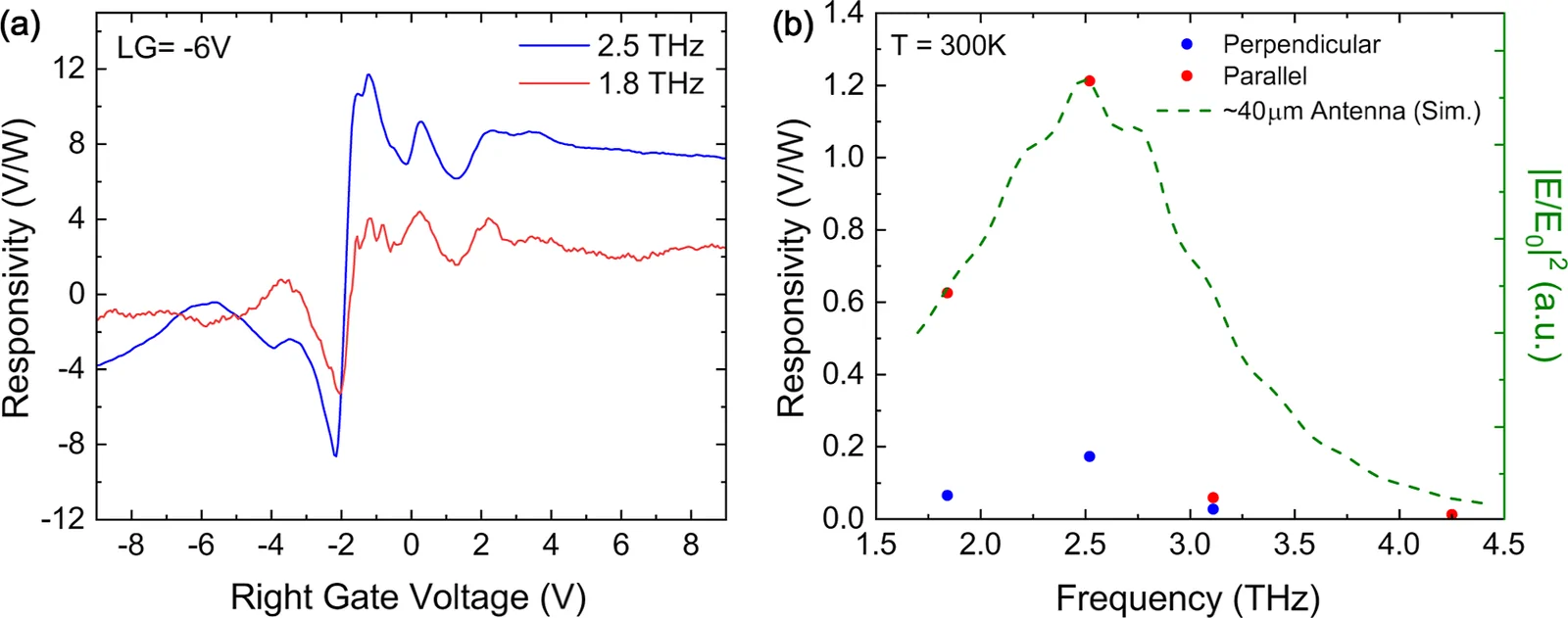
Frequency dependence of device responsivity. (a) Gate-dependent photovoltage measured at *T* = 6 K, *f* = 2.5 THz (blue), and *f* = 1.8 THz (red), showing reduced response at a lower frequency due to weaker antenna coupling. (b) Room-temperature peak responsivity as a function of excitation frequency for incident light-polarized parallel (red symbols) and perpendicular (blue symbols) to the antenna axis. The green dashed line shows simulated normalized near-field enhancement |*E*/*E*_0_|^2^ at the graphene plane (right axis), demonstrating excellent agreement between experiment and simulations. The responsivity maximum coincides with the designed dipole antenna resonance at ∼2.5 THz, confirming that the device performance is dominated by antenna-mediated coupling.

A more systematic analysis is provided in Figure 5b, where we plot the room-temperature extracted peak responsivity as a function of frequency for two orthogonal incident polarizations: parallel (red symbols) and perpendicular (blue symbols) to the antenna main axis. A clear polarization dependence emerges: the responsivity is maximized when the electric field is aligned with the dipole antenna arms, confirming that efficient coupling occurs when the incident field drives the antenna directly. In contrast, when the incident field is polarized perpendicular to the antenna main axis, the photoresponse is strongly suppressed, as the dipolar mode is not properly excited. The overall spectral dependence of the responsivity also agrees well with full-wave simulations of the antenna-coupled device, shown as the green dashed line in Figure 5b. Here, the simulated normalized near-field enhancement |*E*/*E*_0_|^2^ at the graphene plane (right axis) closely follows the measured responsivity trend, with a peak around *f* ≈ 2.5 THz and a rapid decrease at higher and lower frequencies. This consistency confirms that the frequency dependence of the device performance is primarily governed by the antenna resonance, as also supported by the simulated antenna response in Figure S2.

We have demonstrated a resonant THz photodetector that combines a dipole-antenna split gate with a CVD single-layer graphene channel to harness the PTE and AGP modes. Under 2.5 THz illumination at cryogenic temperature, the device exhibits two robust, gate-tunable resonances that coincide with simulated absorption maxima and standing-wave field patterns. The first resonance spans the full channel, whereas the second resonance is predominantly confined beneath the right gate, consistent with a Fabry–Pérot picture in which the carrier-density and conductivity profile along the channel defines the effective cavity length, while mobility asymmetry primarily sets the level of plasmon damping. The photovoltage scales linearly with incident power over nearly two decades, follows the antenna polarization, and peaks at the designed antenna resonance, confirming antenna-mediated coupling as the dominant excitation pathway. As temperature increases, the resonance amplitudes decrease, consistent with enhanced intrinsic plasmon damping arising from thermally activated carrier scattering and screening, for which carrier mobility provides a useful qualitative indicator. At liquid-nitrogen and lower temperatures, the AGP cavity is highly efficient: the first AGP resonance alone induces a relative modulation of the PTE photovoltage signal of up to ∼40%, while the second AGP resonance produces modulations of up to ∼25–30%, demonstrating that the resonant contribution dominates the overall response in the cryogenic regime. The resonances vanish above ∼130 K, consistent with enhanced intrinsic plasmon damping arising from thermally activated carrier scattering and screening. Looking forward, higher-quality graphene channels (e.g., encapsulated CVD graphene), lower-loss dielectrics, and optimized cavity boundaries are expected to reduce intrinsic plasmon losses, increase the quality factor *Q*, and extend operation toward elevated temperatures. As temperature increases, the resonance amplitudes decrease, for which carrier mobility provides a useful qualitative indicator. Achieving room-temperature AGP resonances would require substantially improving the carrier scattering time τ. Based on our *Q*-factor framework (*Q* = ωτ at *f* = 2.5 THz), sustaining *Q* ≈ 2 at room temperature would require τ ∼ 0.1–0.2 ps, corresponding to carrier mobilities of ∼15,000–30,000 cm^2^ V^–1^ s^–1^. This is above the typical ∼1,000–3,000 cm^2^ V^–1^ s^–1^ of CVD graphene on SiO_2_ at room temperature, but within the reach of high-quality encapsulated CVD graphene grown in single crystals by epitaxial approaches.^66^ Improving the cavity reflectivity through optimized contact geometry is also expected to further increase *Q*. Beyond evidencing AGP resonances in potentially scalable, nonencapsulated graphene, our results highlight a practical device architecture in which electrostatic design and antenna engineering jointly define the plasmonic cavity and the thermoelectric readout. Integration with on-chip antennas, impedance-matched readout, and pixelated arrays may enable frequency-selective THz imaging and spectroscopy with low power consumption. More broadly, the platform is compatible with other van der Waals heterostructures and could leverage gain mechanisms (tunneling or superconducting contacts) to approach room-temperature plasmonic THz sensing.

## Experimental Section

### Graphene Growth and Transfer

Monolayer graphene was synthesized by chemical vapor deposition (CVD) on high-purity copper foil (35 μm-thick, Alfa Aesar) using a 4″ Aixtron Black Magic. The growth was carried out under a mixture of methane (CH_4_) and hydrogen (H_2_) gases, with flow rates of 30 sccm of CH_4_, diluted in Ar (0.01%) and 50 sccm H_2_, at a growth temperature of ∼1000 °C and pressure of 25 mbar for 6 h.^67^ After growth, the samples were rapidly cooled to room temperature under a H_2_ atmosphere to preserve monolayer quality. The graphene film was transferred onto highly resistive (ρ > 10,000 Ω·cm) Si substrates purchased from Siegert Wafer GmbH, with a 300 nm thermally grown SiO_2_ layer. We used a standard wet-transfer process.^68^ A thin layer of PMMA (A4–950 K, MicroChem) was spin-coated onto the graphene/Cu stack at 4000 rpm for 40 s. The copper foil was subsequently etched away in an aqueous ammonium persulfate (APS) solution (∼3 g in 150 mL of water), followed by multiple rinsing steps in deionized water to remove etchant residues. The graphene/PMMA membrane was carefully scooped and transferred onto the target substrate, followed by drying at room temperature for 8 h. Finally, PMMA was removed by immersion in acetone for 1 h, followed by a rinse in isopropanol and blow-drying in nitrogen, which leaves monolayer graphene on the target substrate. To verify the quality of the transferred graphene, Raman spectroscopy was performed using a Renishaw inVia confocal Raman microscope equipped with a 532 nm excitation laser and a 100× objective lens, as reported in the Supporting Information, Figure S6. The Raman analysis confirmed the preservation of high-quality monolayer graphene before and after transfer.

### Device Fabrication

Source and drain contacts were patterned using a maskless aligner lithography system (using Heidelberg MLA150, from Heidelberg Instruments Mikrotechnik GmbH). The photoresist used was ECI 3007 (positive tone), which was spin-coated at 4000 rpm for 60 s, followed by a soft bake at 100 °C for 1 min. After exposure, a postexposure bake (PEB) was performed at 110 °C for 1 min before development, followed by thermal evaporation of Ti (5 nm)/Au (25 nm) and lift-off in acetone. The resulting source-drain separation was 2 μm, matching the designed channel width. The graphene channel was subsequently patterned into an H-shape via RIE using an O_2_ plasma, RF power of 3 W, and etch time of 30 s, a geometry chosen to optimize the contact resistance between graphene and the metal electrodes. A ∼40 nm-thick AlO_*x*_ layer was then deposited by ALD using trimethylaluminum (TMA) and water as precursors, at a deposition temperature of 250 °C, forming a uniform high-κ gate dielectric. Finally, the split-gate dipole antenna structure was patterned via EBL with a PMMA 950 K resist film, followed by electron-beam evaporation of Ti (5 nm)/Au (50 nm) and lift-off in acetone. The resulting two antenna lobes act as both efficient THz coupling elements and independent electrostatic gates, separated by a 200 nm gap defined by the EBL resolution limit. The total antenna length is 40 μm, optimized through full-wave electromagnetic simulations (see Supporting Information, Figure S2). The graphene conductivity was included as a bidimensional conductive sheet with a complex conductivity computed at the operating frequency, and with a Fermi energy of ∼0.01 eV (undoped graphene). This ensures that the antenna resonance was optimized in the presence of the undoped graphene channel. In this manner, we optimize the antenna to the target THz frequency, independently of the graphene plasmonic resonances, which are optically enhanced by this metallic resonator. This design enables simultaneous optical coupling and electrostatic control of the graphene channel, facilitating the efficient exploitation of the PTE effect. The results presented in this work are from a single, representative device that was selected based on its transport characteristics (mobility, CNP voltage) to be close to our target design parameters. A full statistical analysis across multiple devices is beyond the scope of this initial demonstration and is left for future work, once the relevant fabrication parameters are further optimized.

### Optoelectronic Setup

We use a continuous-wave THz beam from a gas laser (FIRL 100 from Edinburgh Instruments), delivering maximum output powers in the range of a few tens of milliwatts and at discrete emission lines of 1.83, 2.52, 3.11, and 4.25 THz. The THz laser was modulated at 267 Hz using an optical chopper, and the resulting photovoltage was measured by using a lock-in amplifier (Stanford Research Systems). Polarization analysis confirmed that the emitted radiation was strongly linearly polarized with less than 2% residual unpolarized contribution. The detector was oriented such that the antenna axis was aligned with the polarization axes. The THz beam was focused into the cryostat via a 20 mm focal length lens mounted on a cage system, and the incident power was quantified by using a pyroelectric detector (Gentec-EO) positioned in the optical path.

The sample was mounted in a closed-cycle cryostat (Advanced Research Systems, 4 K base temperature) equipped with an optical access and active temperature control. Polarization rotation was achieved by using wire-grid polarizers (Purewave) in combination with crystalline quartz quarter-wave plates (Tydex). In this configuration, the quarter-wave plate rotated the polarization state of the incident beam, while the subsequent polarizer allowed for the arbitrary selection of the polarization direction. The photoresponse was verified to scale linearly with the incident power.

### Responsivity and NEP Calculation

The external responsivity *R*_v_ is given by^26,32,38^

8 $$R_{v}=\frac{V_{ph}}{P_{eff}}=\frac{V_{ph}}{P_{0}\left(A_{diff}/A_{focus}\right)}$$

where *P*_0_ is the power measured by the commercial power meter, *A*_focus_ is the experimentally measured beam area at the sample plane, and *A*_diff_ is the diffraction-limited spot size. The photovoltage *V*_ph_ is obtained from the output of the lock-in amplifier (*V*_LIA_) using $V_{ph}=\frac{2\pi \sqrt{2}}{4}V_{LIA}$ .^26,32^ Since the experimental beam is much larger than a diffraction-limited spot, only the fraction *A*_diff_/*A*_focus_ of the measured power *P*_0_ can, in principle, be concentrated onto the detector. This defines an effective power *P*_eff_ = *P*_0_(*A*_diff_/*A*_focus_). In our experiments, this fraction is *A*_diff_/*A*_focus_ ≈ 1/290 ≈ 3.4 × 10^–3^. The ratio between the diffraction-limited and experimental areas is

9 $$\frac{A_{diff}}{A_{focus}}=\frac{w_{0,diff}^{2}}{w_{0,x}w_{0,y}}$$

where the beam radii *w*_0,*x*_ and *w*_0,*y*_ are extracted by scanning the device in the *x*- and *y*-directions and fitting the photocurrent profiles with Gaussian functions $\propto e^{-2x^{2}/w_{0,x}^{2}}$ and $\propto e^{-2y^{2}/w_{0,y}^{2}}$ . These radii are related to the standard deviation via σ = *w*_0_/2 and to the full width at half-maximum (FWHM) via $FWHM=\sqrt{2\;\ln2}w_{0}$ . At λ = 120 μm, we typically obtain *w*_0,*x*_ ≈ *w*_0,*y*_ = 0.65 mm (see Figure S5). For the diffraction-limited spot, we use *w*_0,diff_ = λ/π, giving *A*_diff_ = π*w*_0,diff_^2^ = λ^2^/π. Finally, the noise-equivalent power (NEP) is defined as NEP = *V*_noise_/*R*_v,_ and, since the unbiased device exhibits very low intrinsic noise dominated by Johnson noise, we use a noise spectral density $V_{noise}=\sqrt{4k_{B}TR_{D}}$ , where *k*_B_ is the Boltzmann constant, *T* is the operating temperature, and *R*_D_ is the device resistance.

## Supplementary Material



## Notes

Electrical mobility characterization of the graphene channel; antenna optimization via full-wave FDTD simulations; temperature-dependent modulation intensity of the AGP resonances; graphene conductivity profiles for the two resonance conditions; THz beam diameter calibration; Raman spectroscopy of graphene at successive fabrication stages; photovoltage as a function of left-gate voltage; and gate-, temperature-, and power-dependent photovoltage response at *f* = 1.8 THz (PDF)
