# Supplementary material for: Enhanced Terahertz Photoresponse via Acoustic Plasmon Cavity Resonances in Scalable Graphene
Source: ACS Photonics. 2026 Jun 1;13(16):4505–17. doi: 10.1021/acsphotonics.6c00272 (PMC13495942; doi:10.1021/acsphotonics.6c00272)
Supplement: Supplementary file 1 [file ph6c00272_si_001.pdf]

Supplementary Materials to: Enhanced Terahertz Photoresponse via Acoustic Plasmon Cavity Resonances in Scalable Graphene

Domenico De Fazio[1,2,*,†], Sebastián Castilla[2,*,†], Karuppasamy P. Soundarapandian[2], Tetiana Slipchenko[3,4], Ioannis Vangelidis[5], Simone Marconi[2], Riccardo Bertini[2], Vlad Petrica[6], Yang Hao[6], Alessandro Principi[7], Elefterios Lidorikis[5], Roshan K. Kumar[2,8], Luis Martín-Moreno[3,4], and Frank H. L. Koppens[2,9*]

[1] *Department of Molecular Science and Nanosystems, Ca' Foscari University of Venice, Via Torino 155, 30172 Venice, Italy*

[2] *ICFO—Institut de Ciències Fotòniques, The Barcelona Institute of Science and Technology, Avinguda Carl Friedrich Gauss 3, 08860 Castelldefels (Barcelona), Spain*

[3] *Instituto de Nanociencia y Materiales de Aragón (INMA), CSIC–Universidad de Zaragoza, 50009 Zaragoza, Spain*

[4] *Departamento de Física de la Materia Condensada, Facultad de Ciencias, Universidad de Zaragoza, 50009 Zaragoza, Spain*

[5] *Department of Materials Science and Engineering, University of Ioannina, GR-451 10 Ioannina, Greece*

[6] *School of Electronic Engineering and Computer Science, Queen Mary University of London, E1 4FZ, London, United Kingdom*

[7] *Department of Physics and Astronomy, University of Manchester, Oxford Road, M13 9PL Manchester, United Kingdom*

[8] *Catalan Institute of Nanoscience and Nanotechnology (ICN2), BIST and CSIC, Campus UAB, 08193 Bellaterra, Spain*

[9] *ICREA—Institució Catalana de Recerca i Estudis Avançats, 08010 Barcelona, Spain and*

[†] *These authors contributed equally*

Contents: 7 pages, 9 figures, and 0 tables.

ELECTRICAL MOBILITY OF GRAPHENE

Supplementary Fig. S1 shows the electrical characterization of the graphene channel used in this work at $T = 6$ K. The experimental data are fitted using the following model:

$$R = R_0 + \frac{L}{W} \cdot \frac{1}{e\mu\sqrt{n_0^2 + \left(\frac{C_{\text{ox}}(V - V_{\text{CNP}})}{e}\right)^2}}, \tag{S1}$$

where R_0 is the residual contact resistance, $L = W = 2\ \mu$m, and $C_{\text{ox}} = \varepsilon_0\varepsilon_r/t_{\text{ox}}$ is the gate capacitance per unit area. In this work, gating is achieved through the top AlO_x dielectric layer of thickness $t_{\text{ox}} = 40$ nm, with $\varepsilon_0 = 8.85 \times 10^{-12}$ F m^{-1} the vacuum permittivity. Using a (DC) dielectric permittivity of $\varepsilon_r \approx 5$, for ALD-grown AlO_x films [S1], we obtain $C_{\text{ox}} = 9.96 \times 10^{-8}$ F cm^{-2}. This C_{ox} value is used consistently for all mobility extractions.

Panel (a) displays the resistance R as a function of right-gate voltage V_{RG} with the left gate grounded, yielding mobilities of $\mu_e \approx 1.33 \times 10^4\,\text{cm}^2\text{V}^{-1}\text{s}^{-1}$ for electrons and $\mu_h \approx 3.7 \times 10^4\,\text{cm}^2\text{V}^{-1}\text{s}^{-1}$ for holes. Panel (b) reports the corresponding data for the left-gated region (V_{LG} sweep), where we extract $\mu_e \approx 1.16 \times 10^4\,\text{cm}^2\text{V}^{-1}\text{s}^{-1}$ and $\mu_h \approx 2.7 \times 10^4\,\text{cm}^2\text{V}^{-1}\text{s}^{-1}$. Panel (c) shows the resistance when sweeping both gates simultaneously, resulting in symmetric doping across the entire channel; here, the extracted electron mobility is $\mu_e \approx 2.5 \times 10^3\,\text{cm}^2\text{V}^{-1}\text{s}^{-1}$, while hole mobility is $\mu_h \approx 1.0 \times 10^4\,\text{cm}^2\text{V}^{-1}\text{s}^{-1}$, indicating stronger scattering in the central graphene region. The significant difference in mobility between the left and right gate regions is consistent with additional disorder introduced during fabrication, as discussed in the main text.

Furthermore, the fitting procedure also provides estimates for the contact resistance R_0 and the residual carrier density n_0, which are key figures of merit for the device quality. From Figs. S1(a-c), we extract $R_0 \sim 3.3$–5.5 kΩ for the three configurations, consistent with low-resistance Ti/Au contacts optimized through the H-shaped graphene geometry. The extracted residual carrier densities are $n_0 \sim 1.8$–3.6×10^{11} cm^{-2} for the right-gated configuration, $n_0 \sim 2.2$–3.1×10^{11} cm^{-2} for the left-gated case, and up to $n_0 \sim 5.4$–12×10^{11} cm^{-2} when both gates are swept simultaneously. The larger n_0 observed in the uniformly doped case reflects stronger disorder and trapped charges in the channel, which partly explains the degraded mobility.

ANTENNA OPTIMIZATION

The device is optimized by performing optical calculations with the full vector 3D finite-difference time domain (FDTD) method using Lumerical software. We use perfectly matched layer (PML) conditions employed on all boundaries of the computational cell. More information on the photoresponse modelling is described in [S2, S3].

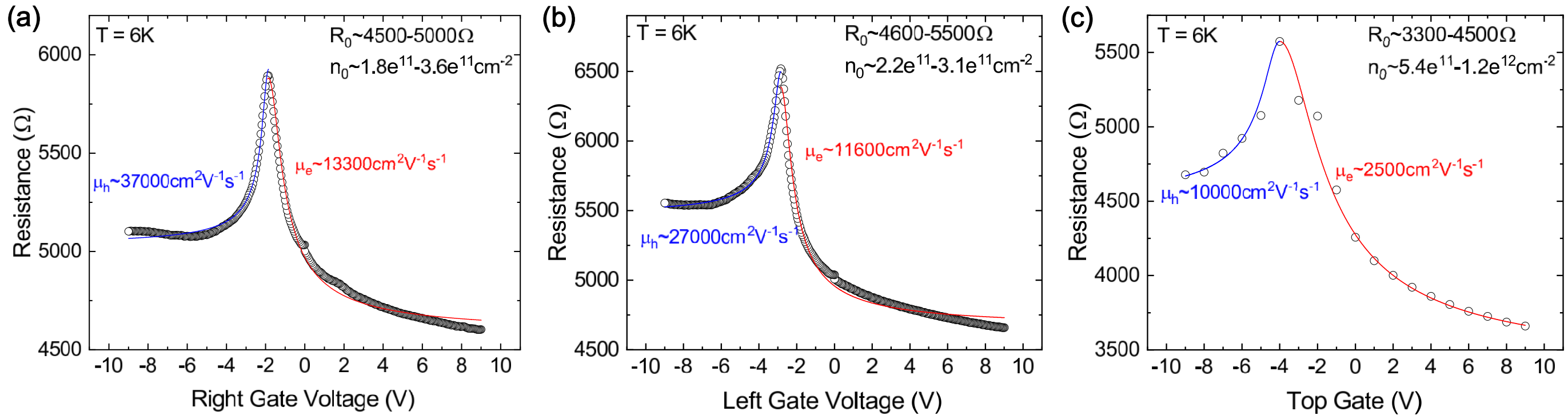

FIG. S1. Low-temperature (T = 6 K) transport characterization of the device. (a) Two-terminal resistance vs. right-gate voltage, yielding $\mu_h \approx 3.7 \times 10^4$ cm^{2}V^{-1}s^{-1} and $\mu_e \approx 1.33 \times 10^4$ cm^{2}V^{-1}s^{-1}. (b) Left-gate sweep, yielding $\mu_h \approx 2.7 \times 10^4$ cm^{2}V^{-1}s^{-1} and $\mu_e \approx 1.16 \times 10^4$ cm^{2}V^{-1}s^{-1}. (c) Uniform gating configuration, showing reduced mobilities $\mu_h \approx 1.0 \times 10^4$ cm^{2}V^{-1}s^{-1} and $\mu_e \approx 2.5 \times 10^3$ cm^{2}V^{-1}s^{-1}. Extracted R_0 and n_0 values are indicated in each panel.

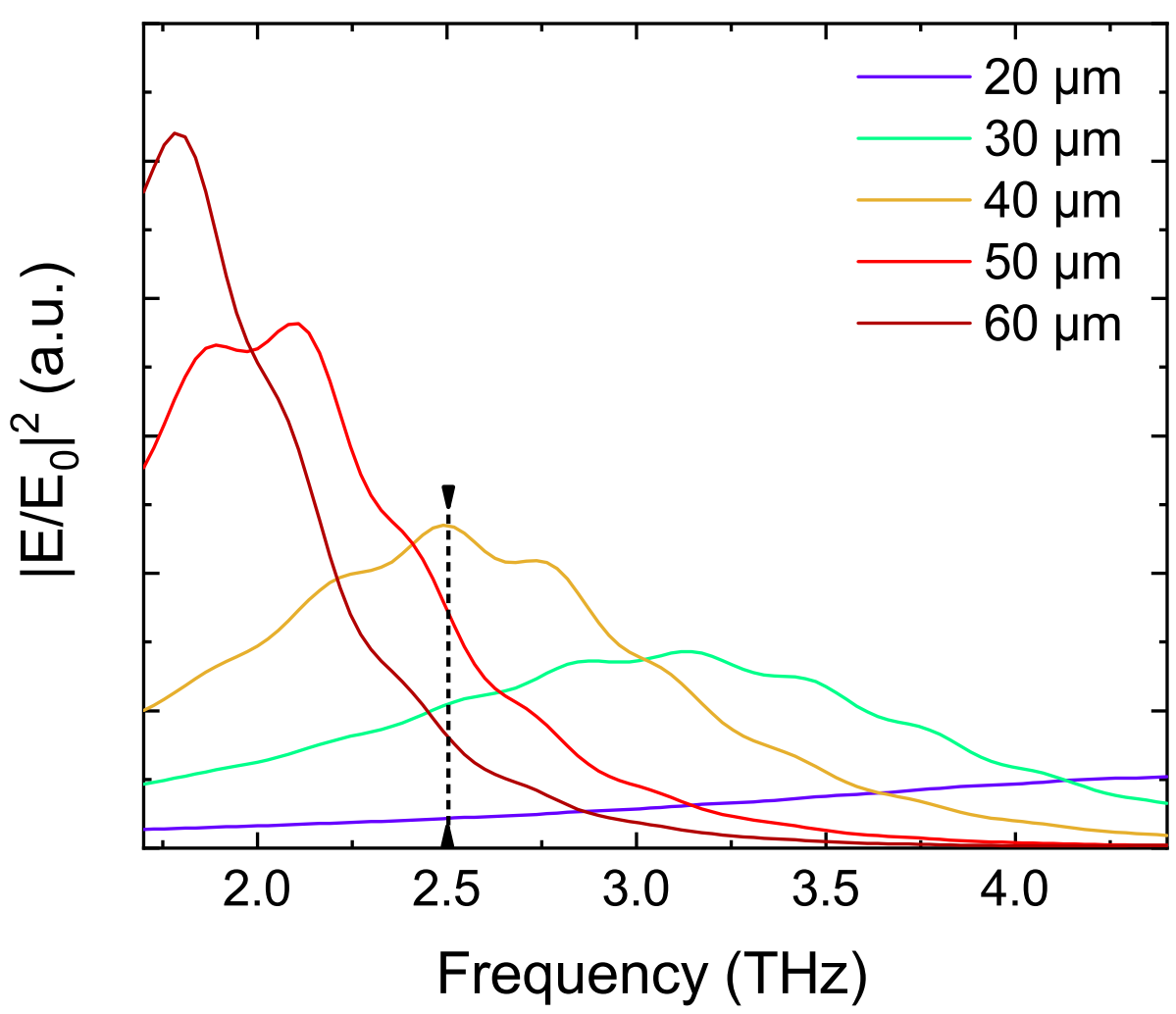

FIG. S2. Field intensity as a function of the incident THz frequency for different dipole antenna lengths. The length includes both branches (left and right) of the antenna and the gap between them. The black arrow indicates the targeted resonance at 2.5 THz, corresponding to the fabricated antenna length of 40 μm.

The stack is made up of two 30 nm thick nanorods separated by a 200 nm gap (the half-wave dipole antenna), a 40 nm thick alumina layer, a graphene layer, a 2 μm thick SiO_2 layer, and a Silicon substrate. The graphene layer is modeled using an H-shaped 2D conductive material having a 2 μm wide central region. The optical properties of the dielectric layers are described by means of a complex permittivity model, while for graphene, a complex conductivity model is used. The excitation source is a Total Field Scattered Field (TFSF) source. The length of the nanorods is swept in the range 20 μm - 60 μm to tune the resonance frequency at the desired value of 2.5 THz. As it is possible to observe in Fig. S2, where the electric field enhancement at the center of the antenna's gap is reported, such a value of resonance frequency is obtained for a length of the nanorods equal to 40 μm.

TEMPERATURE–DEPENDENT MODULATION INTENSITY OF THE AGP RESONANCES

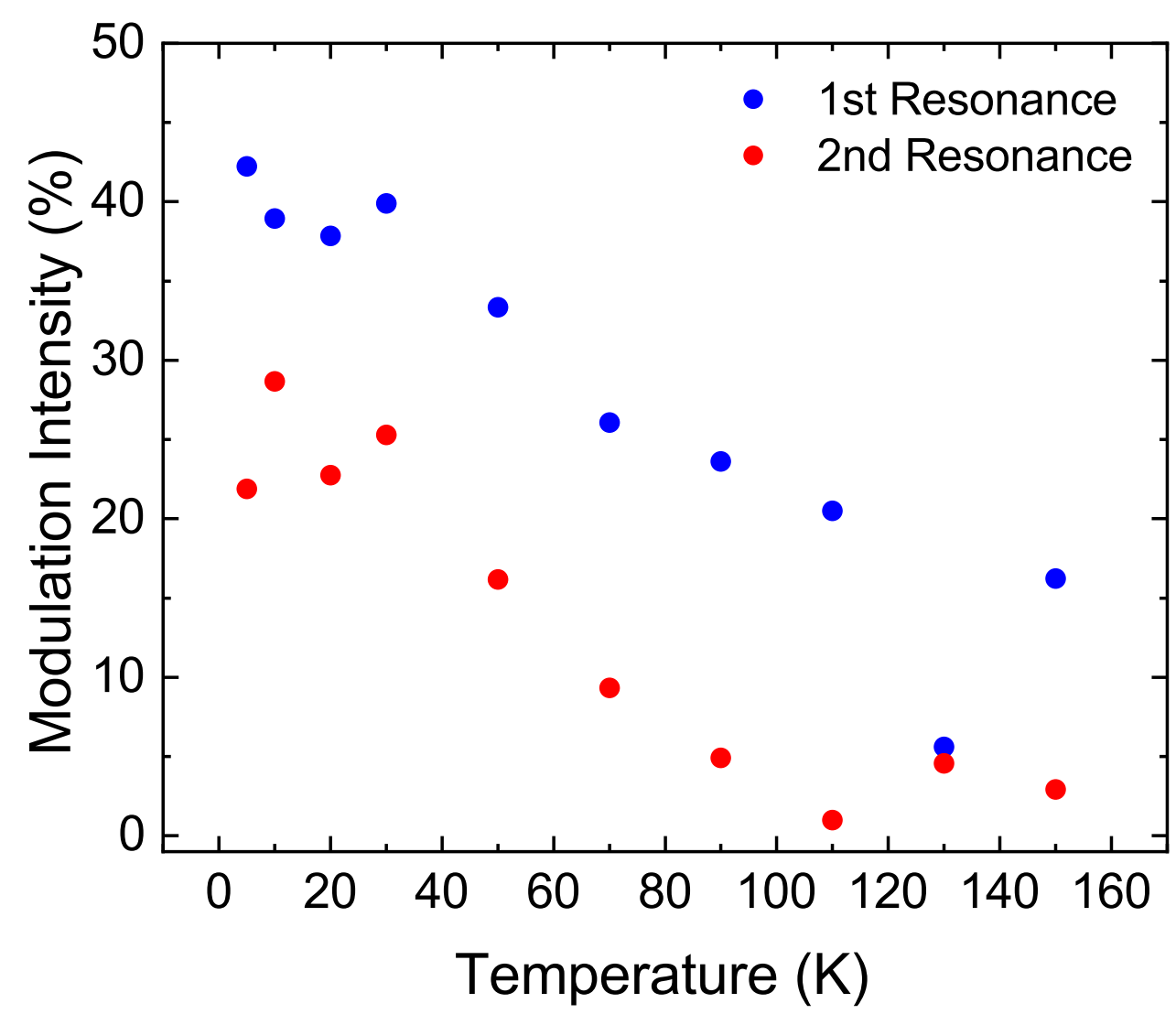

FIG. S3. Modulation intensity $M_i(T)$ of the first (blue symbols) and second (red symbols) AGP resonances, extracted from the right-gate sweeps in Fig. 2(d) of the main text at $V_{\mathrm{LG}} = -6$ V and $f = 2.5$ THz. The modulation intensity is defined as the normalized peak-to-trough contrast between the resonance maximum and the subsequent minimum encountered while sweeping V_{RG} in the same direction [Eq. (S2)].

To quantify how strongly the AGP resonances modulate the PTE response, we introduce a temperature-dependent *modulation intensity* for each resonance. This analysis is based on the right-gate sweeps V_{RG} at fixed $V_{\mathrm{LG}} = -6$ V under $f = 2.5$ THz illumination and for

temperatures between $T = 6$ and 150 K, *i.e.*, on the same dataset used in Fig. 2(d,f) of the main text.

For each temperature T, we consider the absolute photovoltage $|V_{\rm ph}(V_{\rm RG}, T)|$ and identify the local maximum $|V_{\rm ph}^{\max,i}(T)|$ associated with the i-th AGP resonance ($i = 1$ for the 1$^{\rm st}$ resonance and $i = 2$ for the 2$^{\rm nd}$ resonance). Starting from this maximum and continuing the sweep in the same direction of increasing $V_{\rm RG}$, we then locate the nearest local minimum $|V_{\rm ph}^{\min,i}(T)|$ that represents the background response after the system has been driven through the corresponding resonance. We define the modulation intensity of the i-th resonance at temperature T as the normalized peak-to-trough contrast:

$$M_i(T) = \frac{|V_{\rm ph}^{\max,i}(T)| - |V_{\rm ph}^{\min,i}(T)|}{|V_{\rm ph}^{\max,i}(T)|}\,, \quad \text{(S2)}$$

and express $M_i(T)$ in percent. By construction, $M_i(T) = 0$ corresponds to a completely unmodulated response (no discernible resonance), whereas $M_i(T) \to 100\%$ would indicate a fully quenched background between the peak and the nearby minimum. This definition is similar to the contrast metrics commonly used in near-field measurements of graphene plasmons, where the relative modulation of the signal encodes the plasmon quality factor and propagation length [S4].

The extracted modulation intensities for the first and second resonances are summarized in Supplementary Fig. S3. At the lowest temperatures ($T \lesssim 20$ K), the first resonance exhibits a modulation intensity M_1 of approximately 40–45%, while the second resonance reaches $M_2 \approx 25$–30%. As the temperature is increased, both resonances show a monotonic reduction of $M_i(T)$: around $T \approx 90$ K we find $M_1 \approx 25\%$ and $M_2 \approx 10$–15%, whereas at $T \approx 130$–150 K the modulation intensities fall below $\sim 15\%$ and $\sim 5\%$ for the first and second resonance, respectively. This quantitative trend confirms that the AGP resonances provide a substantial modulation of the PTE response at low temperatures and progressively fade as plasmon damping increases. The systematically larger modulation intensity of the first resonance can be understood within the cavity picture developed in the main text. The first mode corresponds predominantly to an AGP standing wave that spans the full channel, with an electric-field maximum near the antenna gap and strong overlap with the region of largest Seebeck asymmetry. In contrast, the second mode is more localized toward the right half of the channel and is therefore more susceptible to intrinsic plasmon losses arising from disorder and spatial variations of the transport properties along the channel, which effectively reduce the cavity quality factor and hence the modulation depth. This behaviour is fully consistent with the overall temperature evolution of the AGP resonances discussed in the main text, where a reduction of carrier mobility by nearly two orders of magnitude between $T = 6$ and 130 K is accompanied by enhanced plasmon damping and the eventual disappearance of the resonant features. More broadly, the temperature dependence of $M_i(T)$ mirrors the trend reported for the plasmonic quality factor $Q = Q(T)$ in cryogenic near-field imaging experiments on high-mobility encapsulated graphene [S4]. In those studies, a rapid increase of Q and of the plasmon propagation length is observed upon cooling, reflecting a dramatic reduction in the intrinsic plasmonic scattering rate. Our modulation-intensity analysis shows that, even in a CVD-grown and non-encapsulated graphene platform, the AGP cavity retains a strong resonant character at liquid-nitrogen and lower temperatures: the first AGP mode alone can modulate the PTE photoresponse by $\sim 40\%$ at $T = 6$ K, highlighting the potential of such structures for plasmonically enhanced THz detection.

CONDUCTIVITY

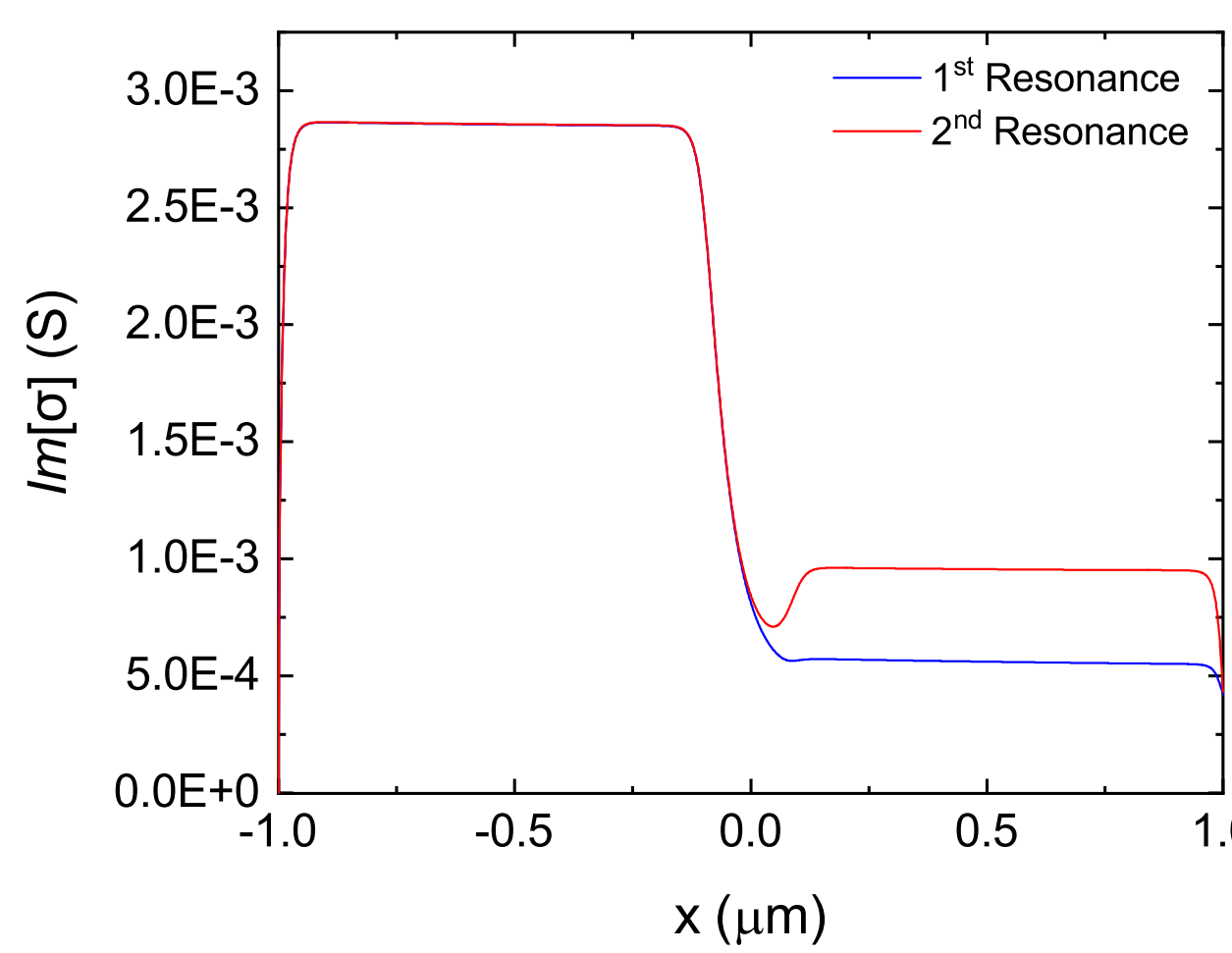

FIG. S4. Imaginary part of the graphene conductivity in the case of the 1$^{\rm st}$ and 2$^{\rm nd}$ resonance.

The reason why, in the case of the 1$^{\rm st}$ resonance, the entire graphene channel participates in the Fabry—Perot mode, whereas for the 2$^{\rm nd}$ resonance, only half of the channel is effectively involved, is non-trivial. Here, we attribute it to the combined effect of graphene mobility asymmetry (under the two gates) and an abrupt change in surface conductivity in the gap (see Fig. S4). The lower mobility under the left gate increases plasmon damping and hinders efficient plasmon propagation in that region, while the abrupt conductivity change in the gap region acts as an additional plasmon reflector. As a result, in the 2$^{\rm nd}$ resonance case, the Fabry–Perot condition is primarily sustained by the higher-mobility right side, yielding a local plasmon wavelength of $\lambda_{\rm p}^{(2)}$ close to the half-channel resonance. By contrast, at the 1$^{\rm st}$ resonance, the effective

plasmon wavelength is determined by both halves of the channel, $\lambda_p^{(1)}$, corresponding to the full-channel Fabry–Perot condition. In this case, the smoother conductivity profile across the channel suppresses plasmon backscattering at the gap region, enabling a standing wave that extends across the entire device despite the higher losses on the left side.

BEAM DIAMETER

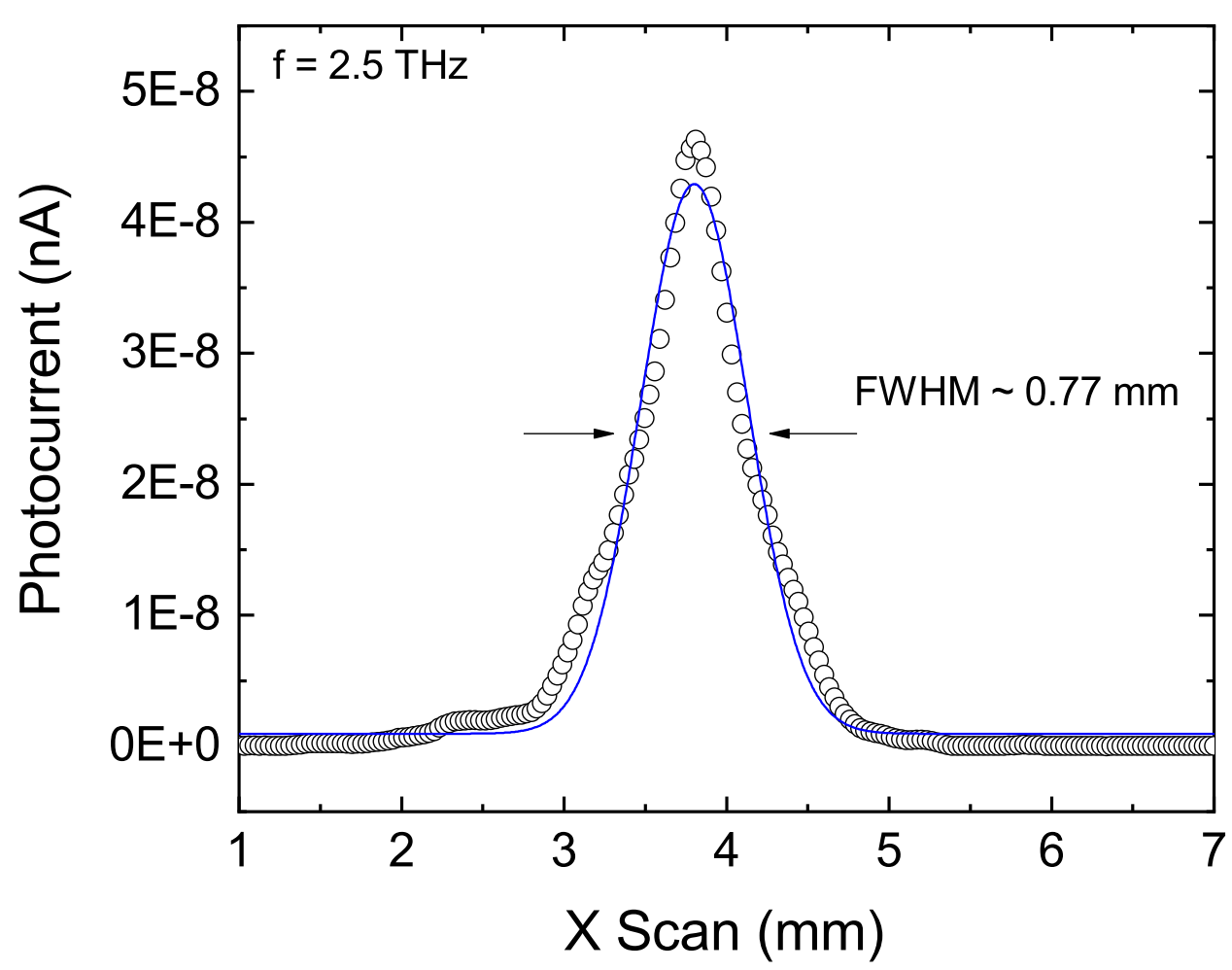

FIG. S5. Determination of the THz beam diameter at the sample position. The photocurrent is measured as a function of lateral beam position (x-scan) at $f = 2.5$ THz. Open symbols represent the experimental data, while the solid line is a Gaussian fit.

To determine the effective beam diameter at the device plane, the THz spot was scanned laterally across the detector using a motorized servo stage. At each position x, the photocurrent was recorded at a fixed frequency of $f = 2.5$ THz. The resulting spatial profile, shown in Supplementary Fig. S5, exhibits a Gaussian shape centered on the device location. Fitting the data with a Gaussian function:

$$I_{\rm ph}(x) = I_0 \exp\left[-\frac{(x-x_0)^2}{2\sigma^2}\right], \quad \text{(S3)}$$

yields FWHM = 0.771 mm ($\sigma = 0.328$ mm, $R^2 \sim 1$). The statistical uncertainty on the fit parameters is negligible; the dominant uncertainty is systematic, estimated at $\lesssim 5\%$ from the reproducibility of the beam scan. Via the quadratic dependence of $A_{\rm focus}$ on w_0, this translates into a systematic uncertainty of $\lesssim 10\%$ in the reported responsivity values, which we treat as the dominant source of error in our responsivity estimates. This FWHM corresponds to a $1/e^2$ beam radius $w_0 = {\rm FWHM}/\sqrt{2\ln 2} \approx 0.65$ mm, consistent with the values $w_{0,x} \approx w_{0,y}$ used in the Experimental Section. The illuminated beam area at the sample is then $A_{\rm focus} = \pi w_{0,x} w_{0,y}$, which we identify with the experimental beam area $A_{\rm focus}$ entering the responsivity definition $R_{\rm v} = V_{\rm ph}/P_{\rm eff} = V_{\rm ph}/[P_0\,(A_{\rm diff}/A_{\rm focus})]$ in the main text.

RAMAN SPECTROSCOPY OF GRAPHENE

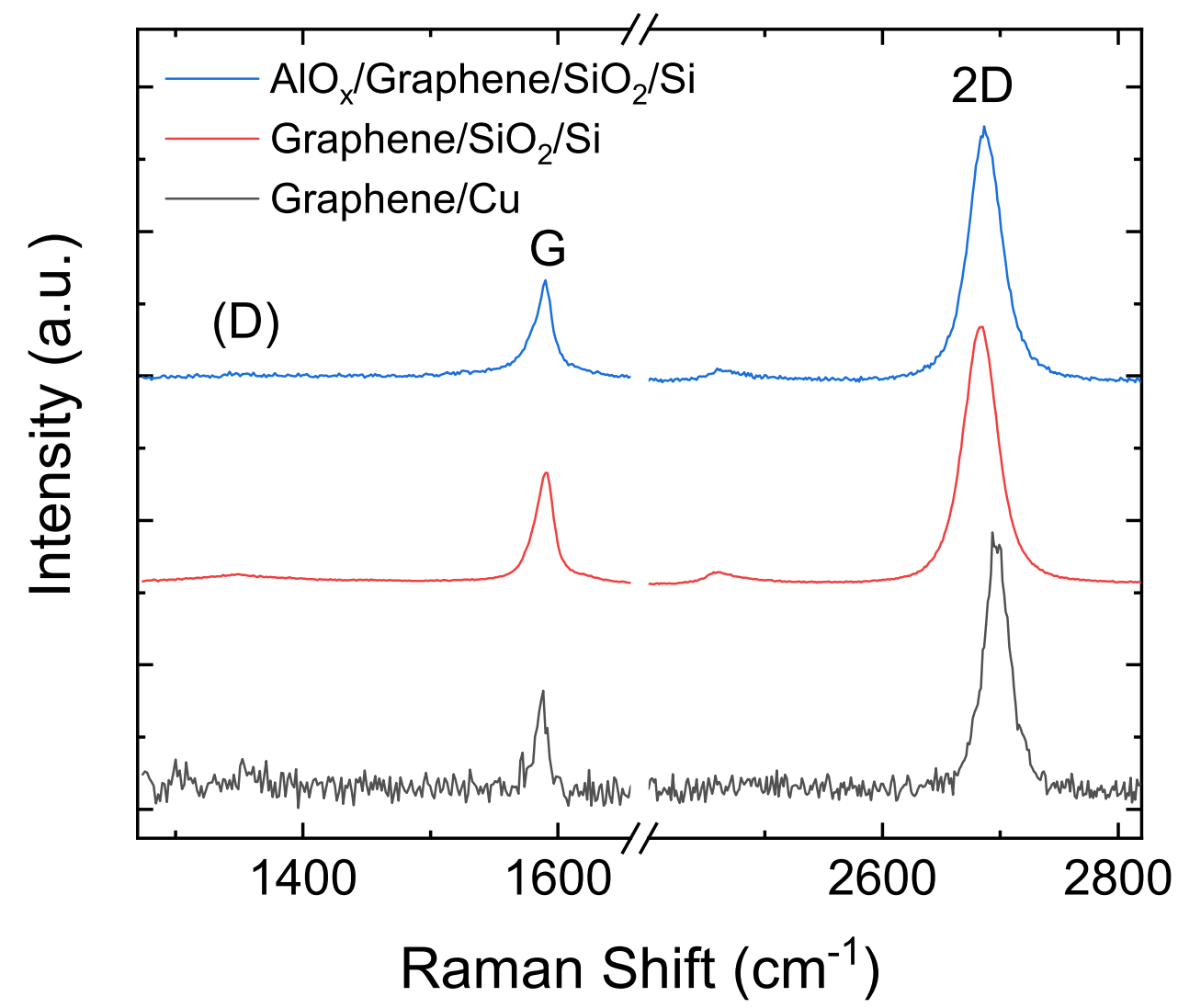

FIG. S6. Raman spectra of graphene at different stages of device fabrication: as-grown on Cu (black), after wet transfer onto SiO_2/Si (red), and after AlO_x deposition (blue). All spectra are normalized to the maximum peak intensity and vertically offset for clarity. Measurements were performed using a Renishaw InVia spectrometer with a 532 nm excitation wavelength and 100× objective, maintaining the incident laser power below 1 mW to prevent sample damage.

Supplementary Fig. S6 shows the Raman spectra of monolayer graphene acquired at different stages of the fabrication process. All measurements were taken with a Renishaw InVia Raman spectrometer equipped with a 100× objective lens and an excitation wavelength of 532 nm, while keeping the laser power below 1 mW to avoid sample heating or degradation. The spectra were normalized to the height of the maximum peak, and for the as-grown graphene on Cu, the broad photoluminescence background from the metallic substrate was subtracted for clarity [S5].

The bottom (black) curve corresponds to graphene directly after CVD growth on Cu foil. The middle (red) curve shows the spectrum after wet transfer onto SiO_2/Si, while the top (blue) curve corresponds to the graphene after AlO_x encapsulation. For graphene on SiO_2/Si, the 2D peak appears at Pos(2D) ~ 2683 cm^{-1} with a full width at half maximum FWHM(2D) ~ 33 cm^{-1}. The G peak is located at Pos(G) ~ 1590 cm^{-1} with

FWHM(G) ~ 17 cm^{-1}. The area ratio A(2D)/A(G) ~ 4.6, while the intensity ratio I(2D)/I(G) ~2.4. These values are consistent with monolayer graphene exhibiting a single-Lorentzian 2D peak profile and a ~200-250 meV doping [S6–S9]. Upon AlO_x deposition, the Raman peaks remain sharp and well-defined. The G peak appears at Pos(G) ~ 1589 cm^{-1} with FWHM(G) ~ 18 cm^{-1}, while the 2D peak is centered at Pos(2D) ~ 2687 cm^{-1} with FWHM(2D) ~ 32 cm^{-1}. The intensity and area ratios are I(2D)/I(G) ~ 2.6 and A(2D)/A(G) ~ 5.1, respectively. These values are in line with a ~200-250 meV n-type doping, corroborating our electrical measurements [S6–S9]. Besides, they indicate that the AlO_x deposition induces only minor variations, without any evidence of structural degradation or defect formation. No measurable D peak at ~ 1350 cm^{-1} is observed in any of the spectra, indicating that neither the transfer nor the dielectric deposition introduces additional defects within the detection limit [S6, S8–S10]. Overall, the Raman analysis confirms that the graphene retains its monolayer character and high crystalline quality throughout the full device fabrication sequence.

PHOTOVOLTAGE AS A FUNCTION OF LEFT-GATE VOLTAGE

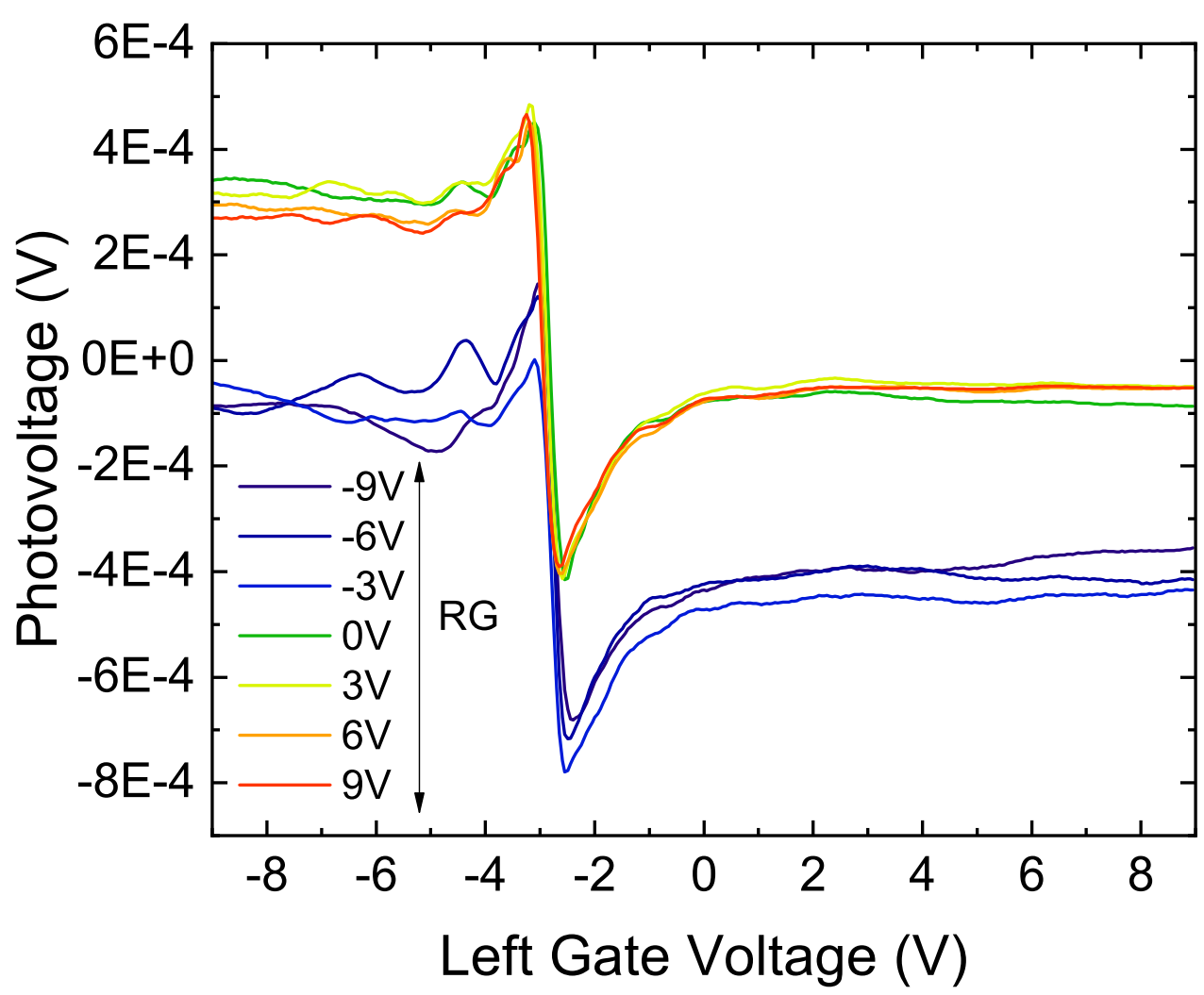

FIG. S7. Photovoltage V_{ph} as a function of left-gate voltage V_{LG} for several fixed right-gate voltages V_{RG} at $T = 6$ K. The curves correspond to $V_{\mathrm{RG}} = -9$ V to $+9$ V in 3 V steps, as indicated by the color scale.

Supplementary Fig. S7 shows the photovoltage V_{ph} measured as a function of left-gate voltage V_{LG} for several fixed values of the right-gate voltage V_{RG}, recorded at $T = 6$ K under 2.5 THz illumination. The measurements reveal that the overall photoresponse remains qualitatively similar to the right-gate sweeps reported in the main text, exhibiting a pronounced polarity inversion around the charge neutrality point (CNP) and multiple smaller oscillatory features at gate voltages below the CNP. Compared to the right-gate dependence shown in Fig. 1 (h) of the main text, the magnitude of the photovoltage modulation with V_{LG} is reduced, and the detailed resonance structure becomes less pronounced. This is expected since the left gate primarily affects the overall Seebeck asymmetry between the two graphene regions, rather than directly tuning the carrier density and plasmonic resonance conditions in the active right-gated section.

These results further corroborate the interpretation discussed in the main text: the right gate controls the formation and spectral position of the AGP resonances, while the left gate plays a secondary role by setting the background doping and thermoelectric contrast required for efficient PTE detection. This dataset is included here for completeness.

PHOTOVOLTAGE RESPONSE AT 1.8 THZ

Supplementary Fig. S8 summarizes the device characterization under illumination at a lower excitation frequency of $f = 1.8$ THz. This measurement serves as a comparative study to the 2.5 THz data presented in the main text, illustrating the response of the same device when the antenna is operated off its designed resonance frequency. Overall, the observed signals confirm the persistence of the PTE response and the presence of gate-dependent modulations associated with plasmonic effects, although with reduced amplitude and increased noise. Panel (a) displays the photovoltage V_{ph} as a function of right-gate voltage V_{RG} for several fixed left-gate voltages V_{LG}. The polarity inversion around the charge neutrality point (CNP) remains visible, consistent with PTE-dominated behavior. Ripple-like oscillations and weaker resonant features appear on the electron-doped side, with broad maxima around $V_{\mathrm{RG}} \approx 0.2$ V and 1.2 V. These features resemble the 1st and 2nd AGP resonances discussed at 2.5 THz, but the lower excitation frequency and off-resonant antenna coupling make their precise identification more uncertain. Panel (b) shows complementary left-gate sweeps at different fixed V_{RG} values. The qualitative dependence is similar to that observed at higher frequency, although the modulation depth with V_{LG} is smaller, indicating weaker sensitivity of the PTE response to left-gate tuning under off-resonant excitation. Panel (c) provides a two-dimensional color map of V_{ph} as a function of both gate voltages at $T = 6$ K, reproducing the sixfold symmetry typical of dual-gated graphene detectors. The contrast is reduced compared to the 2.5 THz case, and the fine oscillatory structure is less pronounced due to the weaker coupling efficiency of the antenna at this frequency.

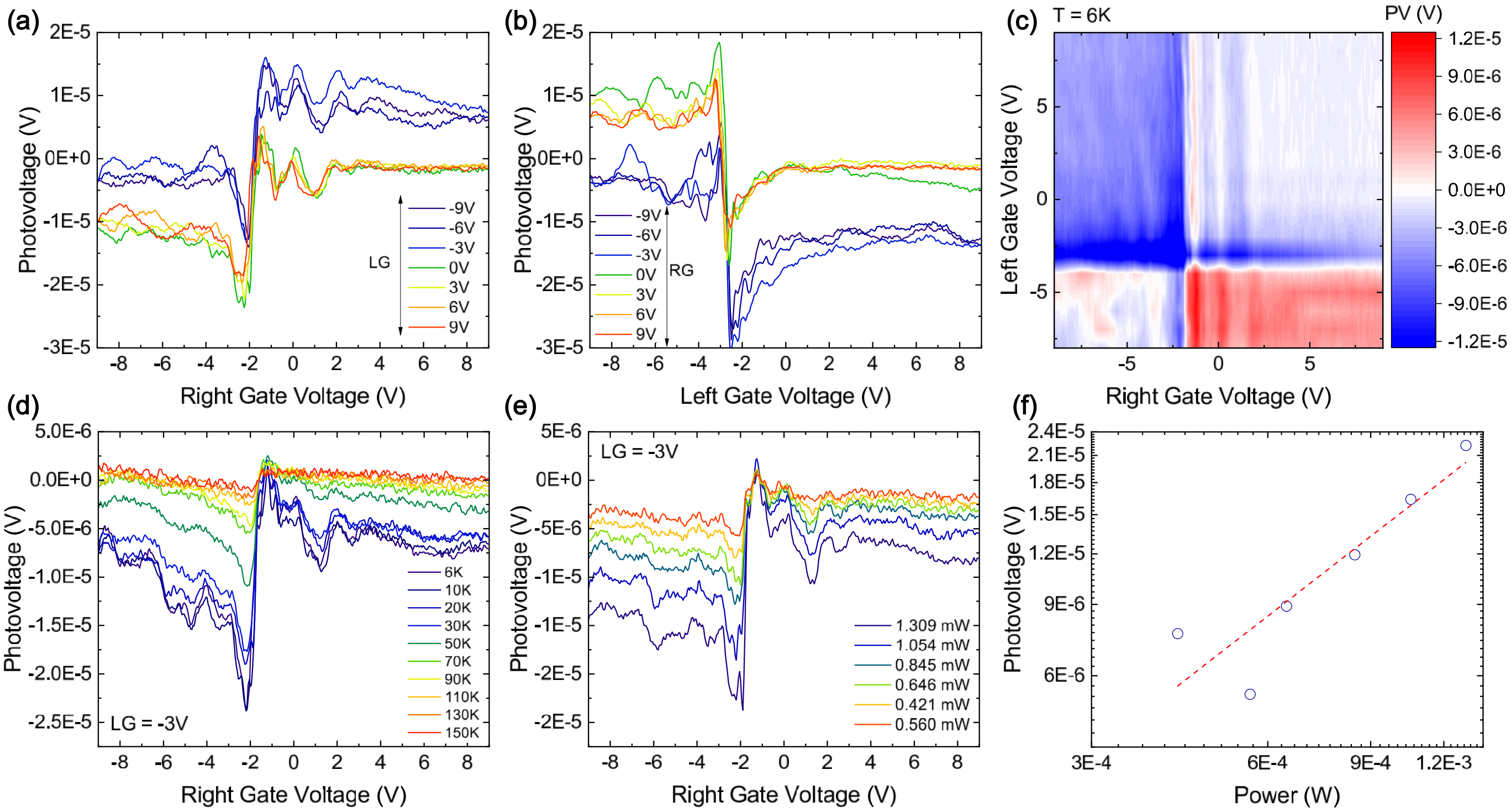

FIG. S8. Gate-, temperature-, and power-dependent photovoltage measurements at $f = 1.8$ THz. (a) Photovoltage V_{ph} as a function of right-gate voltage V_{RG} for several fixed left-gate voltages V_{LG}. (b) Photovoltage as a function of V_{LG} for fixed V_{RG}. (c) Two-dimensional map of $V_{\mathrm{ph}}(V_{\mathrm{LG}}, V_{\mathrm{RG}})$ at $T = 6$ K. (d) and (e) Temperature- and power-dependent right-gate sweeps, respectively. (f) Peak photovoltage as a function of incident power, confirming a linear dependence.

The temperature dependence is examined in panel (d), where the amplitude of the photovoltage progressively decreases with increasing T, consistent with enhanced plasmon damping and reduced carrier mobility. The resonant-like modulations remain visible at low temperature but gradually fade above ~ 100 K. Panel (e) shows the effect of varying the incident THz power, confirming that the overall signal scales monotonically with excitation intensity, even in the off-resonant regime. Finally, panel (f) plots the extracted photovoltage peak amplitude versus incident power on a linear scale, yielding an approximately linear relationship. This behavior confirms that the detector continues to operate in the linear response regime at 1.8 THz, despite the lower signal-to-noise ratio. The results in Supplementary Fig. S8 demonstrate that the device maintains a measurable PTE response and weak plasmonic modulations at 1.8 THz. However, the reduced photovoltage amplitude and increased noise level are consistent with operation away from the antenna resonance, as predicted by electromagnetic simulations (see main text Fig. 5(b)). These measurements further validate the robustness of the detection mechanism over a range of excitation frequencies, even though the plasmonic enhancement is optimized around 2.5 THz.

PHOTOVOLTAGE RESPONSE OF A PRELIMINARY DEVICE

Supplementary Fig. S9 shows the gate-dependent photovoltage response of a preliminary graphene THz detector measured at low and elevated temperatures. Although this device was not used for the detailed analysis presented in the main text, the low-temperature trace exhibits pronounced oscillatory features in the photovoltage as a function of gate voltage, which are qualitatively consistent with plasmonic resonances. These features are strongly suppressed at elevated temperature, in agreement with the temperature-dependent damping behavior discussed for the representative device in the main text. This preliminary measurement, therefore, provides additional qualitative evidence that the observed resonant photoresponse is reproducible across devices, while a full device-to-device statistical analysis remains beyond the scope of the present work.

* domenico.defazio@unive.it; sebastian.castilla@icfo.eu; frank.koppens@icfo.eu

[S1] J. Kolodzey, E. Chowdhury, T. Adam, G. Qui, I. Rau, J. Olowolafe, J. Suehle, and Y. Chen, Electrical conduc-

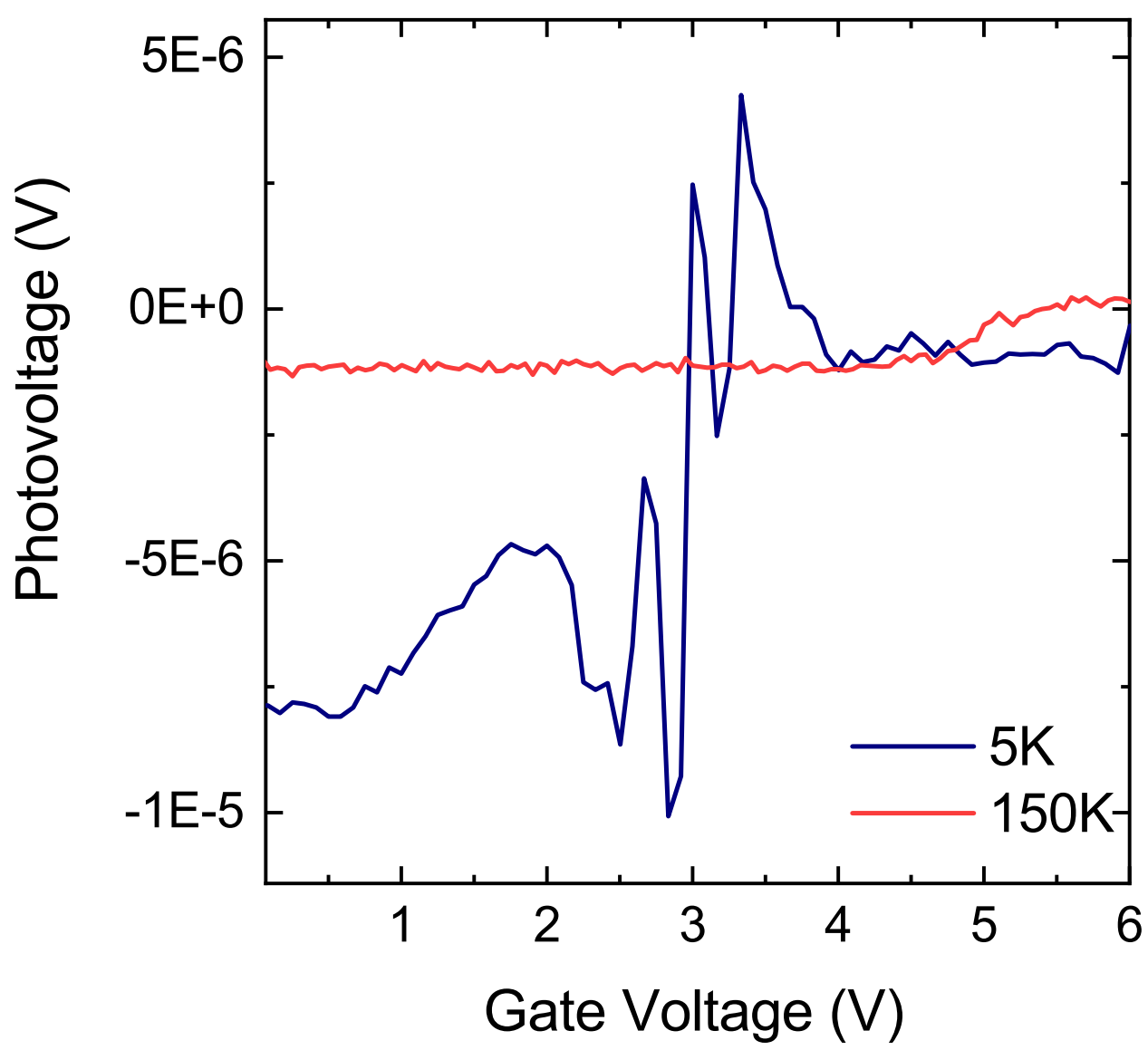

FIG. S9. Gate-dependent photovoltage response of a preliminary graphene THz detector measured at low and elevated temperatures. The blue and red curves correspond to measurements at 5 K and 150 K, respectively. Pronounced oscillatory features are observed at low temperature and are strongly suppressed at elevated temperature, qualitatively consistent with the plasmonic resonance behavior of the device discussed in the main text.

tion and dielectric breakdown in aluminum oxide insulators on silicon, IEEE Trans. Electron. Devices **47**, 121 (2000).

[S2] S. Castilla, I. Vangelidis, V.-V. Pusapati, J. Goldstein, M. Autore, T. Slipchenko, K. Rajendran, S. Kim, K. Watanabe, T. Taniguchi, L. Martín-Moreno, D. Englund, K.-J. Tielrooij, R. Hillenbrand, E. Lidorikis, and F. H. L. Koppens, Plasmonic antenna coupling to hyperbolic phonon-polaritons for sensitive and fast mid-infrared photodetection with graphene, Nat. Commun. **11**, 4872 (2020).

[S3] I. Vangelidis, D. V. Bellas, S. Suckow, G. Dabos, S. Castilla, F. H. L. Koppens, A. C. Ferrari, N. Pleros, and E. Lidorikis, Unbiased Plasmonic-Assisted Integrated Graphene Photodetectors, ACS Photonics **9**, 1992 (2022).

[S4] G. X. Ni, A. S. McLeod, Z. Sun, L. Wang, L. Xiong, K. W. Post, S. S. Sunku, B.-Y. Jiang, J. Hone, C. R. Dean, M. M. Fogler, and D. N. Basov, Fundamental limits to graphene plasmonics, Nature **557**, 530 (2018).

[S5] A. A. Lagatsky, Z. Sun, T. S. Kulmala, R. S. Sundaram, S. Milana, F. Torrisi, O. L. Antipov, Y. Lee, J. H. Ahn, C. T. A. Brown, W. Sibbett, and A. C. Ferrari, 2 μm solid-state laser mode-locked by single-layer graphene, Appl. Phys. Lett. **102**, 013113 (2013).

[S6] A. C. Ferrari, J. C. Meyer, V. Scardaci, C. Casiraghi, M. Lazzeri, F. Mauri, S. Piscanec, D. Jiang, K. S. Novoselov, S. Roth, and A. K. Geim, Raman Spectrum of Graphene and Graphene Layers, Phys. Rev. Lett. **97**, 187401 (2006).

[S7] A. C. Ferrari and D. M. Basko, Raman spectroscopy as a versatile tool for studying the properties of graphene, Nat. Nanotechnol. **8**, 235 (2013).

[S8] A. Das, S. Pisana, B. Chakraborty, S. Piscanec, S. K. Saha, U. V. Waghmare, K. S. Novoselov, H. R. Krishnamurthy, A. K. Geim, A. C. Ferrari, and A. K. Sood, Monitoring dopants by Raman scattering in an electrochemically top-gated graphene transistor, Nat. Nanotechnol. **3**, 210 (2008).

[S9] D. M. Basko, S. Piscanec, and A. C. Ferrari, Electron-electron interactions and doping dependence of the two-phonon Raman intensity in graphene, Phys. Rev. B **80**, 165413 (2009).

[S10] L. G. Cançado, A. Jorio, E. H. M. Ferreira, F. Stavale, C. A. Achete, R. B. Capaz, M. V. O. Moutinho, A. Lombardo, T. S. Kulmala, and A. C. Ferrari, Quantifying Defects in Graphene via Raman Spectroscopy at Different Excitation Energies, Nano Lett. **11**, 3190 (2011).
